# Temperature response of bundle-sheath conductance in maize leaves

**DOI:** 10.1093/jxb/erw104

**Published:** 2016-03-11

**Authors:** Xinyou Yin, Peter E.L. van der Putten, Steven M. Driever, Paul C. Struik

**Affiliations:** Centre for Crop Systems Analysis, Department of Plant Sciences, Wageningen University, PO Box 430, 6700 AK Wageningen, The Netherlands

**Keywords:** Diffusive resistance, maximum PEPc activity, maximum Rubisco activity, modelling, warming effect, *Zea mays*.

## Abstract

A small bundle-sheath conductance (*g*_bs_) is essential for the C_4_ CO_2_-concentrating mechanism to suppress photorespiration effectively. To predict the productivity of C_4_ crops accurately under global warming, it is necessary to examine whether and how *g*_bs_ responds to temperature. We investigated the temperature response of *g*_bs_ in maize by fitting a C_4_ photosynthesis model to combined gas exchange and chlorophyll fluorescence measurements of irradiance and CO_2_ response curves at 21% and 2% O_2_ within the range of 13.5–39 °C. The analysis was based on reported kinetic constants of C_4_ Rubisco and phospho*enol*pyruvate carboxylase and temperature responses of C_3_ mesophyll conductance (*g*_m_). The estimates of *g*_bs_ varied greatly with leaf temperature. The temperature response of *g*_bs_ was well described by the peaked Arrhenius equation, with the optimum temperature being ~34 °C. The assumed temperature responses of *g*_m_ had only a slight impact on the temperature response of *g*_bs_. In contrast, using extreme values of some enzyme kinetic constants changed the shape of the response, from the peaked optimum response to the non-peaked Arrhenius pattern. Further studies are needed to confirm such an Arrhenius response pattern from independent measurement techniques and to assess whether it is common across C_4_ species.

## Highlight

Bundle-sheath conductance, *g*_bs_ , is commonly assumed to be independent of temperature. We report that temperature response of maize *g*_bs_ followed a peaked or non-peaked Arrhenius equation, triggering further investigations on this response.

## Introduction

C_4_ crop species have the CO_2_-concentrating mechanism (CCM) in photosynthesis, which raises the partial pressure of CO_2_ in bundle-sheath cells to a very high level, thereby minimizing the oxygenation activity of Rubisco and the loss by photorespiration (Hatch *et al.*, 1995). This explains why C_4_ food crops such as maize (*Zea mays* L.) generally have higher productivities than their C_3_ counterparts (Ort and Long, 2014), at least under relatively warm conditions, and why C_4_ species are preferred as a major source of sustainable bioenergy production (Heaton *et al.*, 2008; Slattery and Ort, 2015).

The CCM mechanism in C_4_ crops requires a number of biochemical, physical, and structural adaptations, especially the Kranz anatomy including the specialization of the mesophyll and bundle-sheath cells (Hatch *et al.*, 1995; Leegood, 2002; Kromdijk *et al.*, 2014). In C_4_ photosynthesis, CO_2_ is first fixed via phospho*enol*pyruvate carboxylase (PEPc) in mesophyll cells into C_4_ acids, which are then transported into bundle-sheath cells where the C_4_ acids are decarboxylated and the released CO_2_ is refixed by Rubisco. An efficient CCM would require that (i) PEPc has higher CO_2_ affinity and carboxylation capacity than Rubisco; and (ii) the rate of CO_2_ leakage from bundle-sheath cells back into mesophyll cells (*L*) is low.

This CO_2_ leakage rate *L* depends on both the bundle-sheath conductance for CO_2_ (*g*_bs_) and the gradient between the CO_2_ concentration in mesophyll cells (*C*_m_) and that around the carboxylation sites in bundle-sheath cells (*C*_c_): *L*=*g*_bs_(*C*_c_ *–C*_m_) (von Caemmerer and Furbankm 1999; Kromdijk *et al.*, 2014). Therefore, *g*_bs_ is an important parameter for the effectiveness of CCM. We have previously analysed how *g*_bs_ responds to nitrogen supply, and showed that *g*_bs_ increases with increasing leaf nitrogen content (Yin *et al.*, 2011)—a trend similar to that found for mesophyll conductance (*g*_m_) in response to leaf photosynthetic capacity in C_3_ photosynthesis (e.g. Loreto *et al.*, 1992). It has been well established that *g*_m_ in C_3_ photosynthesis responds to leaf temperature (Bernacchi *et al.*, 2002; Warren and Dreyer, 2006; Yamori *et al.*, 2006; Scafaro *et al.*, 2011; Evans and von Caemmerer, 2013; Walker *et al.*, 2013), although the effects differ greatly among species (von Caemmerer and Evans, 2015). Massad *et al.* (2007) have parameterized temperature dependence of some C_4_ parameters such as the maximum rates of PEPc carboxylation (*V*_pmax_), of Rubisco carboxylation (*V*_cmax_), and of electron transport. However, there is hardly any information in the literature on whether and how *g*_bs_ responds to leaf temperature. Reports of Kubien *et al.* (2003) and von Caemmerer *et al.* (2014) on the temperature effect on leakiness (ϕ; which is defined as *L*/*V*_p_, where *V*_p_ is the PEPc carboxylation rate) indirectly suggest that *g*_bs_, among many C_4_ parameters, may (co-)vary with temperature. To the best of our knowledge, only one study (Kiirats *et al.*, 2002) has directly reported on the temperature response of *g*_bs_: *g*_bs_ increased almost linearly with increasing leaf temperature within the range of 16–35 °C. That study used a PEPc mutant of *Amaranthus edulis* with a defective C_4_ cycle. However, concerns have been raised about potential alterations of Rubisco kinetic constants, gas diffusion resistance, and bundle-sheath cell structure by the PEPc mutation (He and Edwards, 1996).

Given the effect of elevating atmospheric CO_2_ and global warming, it is necessary to assess the production potential of C_4_ species, as well as whether their relative advantages over C_3_ species vary, under climate change. Like the widely used C_3_ photosynthesis model of Farquhar *et al.* (1980), the C_4_ biochemical models (Berry and Farquhar, 1978; von Caemmerer and Furbank, 1999) or their variants (Collatz *et al.*, 1992; Chen *et al.*, 1994; Yin and Struik, 2009a) are now increasingly coupled with stomatal conductance models and applied to a general ecosystem or crop simulation framework. However, these modelling studies all assume that *g*_bs_ does not vary with temperature, even when applied to a wide range of natural field environments. To apply the biochemical C_4_ photosynthesis model to assess the (relative) production potentials and their response to climate change variables, information on the effects of temperature on *g*_bs_ is urgently needed. The objectives of this study are to assess whether or not *g*_bs_ in leaves of a maize cultivar responds to leaf temperature and, if so, to quantify the magnitude of this effect over a wide range of temperatures. To that end, we use the method of Yin *et al.* (2011) that can estimate *g*_bs_ by fitting a C_4_ photosynthesis model to a wide range of data covering different amounts of photorespiration.

## Materials and methods

### Experimental set-up

An experiment was conducted in a glasshouse at Wageningen University, using maize cv. ‘Atrium’. To spread out the measurement work in time, a weekly staggered sowing was carried out on 28 August and 4, 10, and 18 September 2014, respectively, to grow plants for four replicate measurements. Plants were transplanted 7 d after sowing to pots of 12 litres, with one plant per pot. The initial soil nutrient contents were: 1.23g of nitrogen, 0.69g of phosphate, and 2.49g of potassium per pot. Extra nutrients came from 10.3g per pot of a slow-release fertilizer ‘Osmocote Pro’ (which contained 17% N, 11% P_2_O_5_, 10% K_2_O, 2% MgO plus trace elements). Temperature in the glasshouse was 27±2 °C for daytime and 21±1 °C for night-time . Photoperiod was maintained at 12h d^−1^ (8:00–20:00h), and relative humidity ranged between 60% and 70%. Of the photosynthetically active radiation incident on the glasshouse, 60% was transmitted to plant level. During daytime, supplemental light from 600W HPS Hortilux Schréder lamps (Monster, The Netherlands; 0.4 lamps m^−2^) was switched on automatically as soon as the global solar radiation incident on the glasshouse dropped below 400W m^−2^, and then switched off if it exceeded 500W m^−2^. The supplementary light largely ensured that, despite the staggered sowing, plants for measurements grew under similar light intensities.

### Photosynthesis measurements

After growth in the glasshouse for ~6 weeks, plants were moved to a climate room illuminated by cool-white fluorescent tubes (~350 μmol m^−2^ s^−1^ at leaf level), where all measurements were undertaken. We used an LI-6400XT open gas exchange system with an integrated fluorescence chamber head, enclosing 2cm^2^ areas, for combined gas exchange and chlorophyll fluorescence measurements, which were done on fully expanded leaves at the seventh leaf layer counted from below. The CO_2_ response curves were taken under both 21% and 2% O_2_ conditions, and the ambient CO_2_ (*C*_a_) steps were: 370, 200, 100, 85, 70, 55, 370, 370, 370, 500, 700, and 1500 μmol mol^−1^ (~4min per step) while keeping incident irradiance (*I*_inc_) at 1500 μmol m^−2^ s^−1^ (while measurements were done four times at 370 μmol mol^−1^, data of only the first and fourth times were included for the analysis). For light response curves, *I*_inc_ was of the order of 2000, 1500, 1000, 500, 200, 100, 80, 60, 40, and 20 μmol m^−2^ s^−1^ (~8min per step), while keeping *C*_a_ either at 250 μmol mol^−1^ for 21% O_2_ or at 1000 μmol mol^−1^ for 2% O_2_ conditions; this was done to induce different levels of photorespiration. Gas from a cylinder containing a mixture of N_2_ and required O_2_ was humidified and supplied via an overflow tube to the air inlet of the LI-6400XT where CO_2_ was blended with the gas, and the IRGA calibration was adjusted for O_2_ composition of the gas mixture according to the manufacturer’s instructions.

Each curve was made at six set-point leaf temperatures (13.5, 18, 25, 30, 34, and 39 °C), of which extreme temperatures (13.5, 34, and 39 °C) were achieved not only by setting the temperature in the LI-6400XT measuring head but also by adjusting the temperature of the climate room. Measurement of one replicate took 5 d and was done on the same leaf for all temperatures. Any influence of different measuring days was minimized by randomizing temperatures and measuring days among the replicates. In total, 48 (i.e. 6 temperatures×4 replicates×2 O_2_ levels) light response curves and 48 CO_2_ response curves were generated. Leaf-to-air vapour pressure difference increased with leaf temperature, but was always within the range of 0.5–2.0 kPa, within which little impact of vapour pressure difference on stomatal conductance is expected (Bernacchi *et al.*, 2002). The measurement flow rate was 400 μmol s^−1^. CO_2_ exchange rates were corrected for CO_2_ leakage into and out of the leaf cuvette, based on measurements at specific temperatures using the same flow rate on boiled leaves across a range of CO_2_ levels, and intercellular CO_2_ levels (*C*_i_) were then re-calculated.

For each step of light or CO_2_ response, when the CO_2_ exchange rate reached steady state, steady-state fluorescence (*F*_s_) was measured. Dwyer *et al.* (2007) have shown that for C_4_ leaves, the multiple-flash method is more reliable than the traditional single-flash method in measuring the maximum fluorescence (*F*_m_′). The *F*_m_′ was therefore obtained from using the multiphase flashes: the flash intensity was ~8000 µmol m^−2^ s^−1^ during phase 1 for a duration of 300ms, was attenuated by 35% during phase 2 for ~300ms, and was back to ~8000 µmol m^−2^ s^−1^ for phase 3 of 300ms. The intercept of the linear regression of fluorescence yields against the inverse of the flash intensity during phase 2 gives the estimate of *F*_m_′ (Loriaux *et al.* 2013). The apparent operating efficiency of photosystem II (PSII) e^−^ transport was obtained as: ∆*F*/*F*_m_′=(*F*_m_′–*F*_s_)/*F*_m_′ (Genty *et al.*, 1989; Schreiber *et al.*, 1995).

The portions of the leaf used for above measurements were excised afterwards, with a punch that produced a disc of ~5cm^2^ per leaf portion. The leaf discs were then weighed after drying at 70 °C to constant weight, and total N content was analysed using an element C/N analyser (Flash 2000, Thermo Scientific) based on the Micro-Dumas combustion method.

### Modelling

The model of von Caemmerer and Furbank (1999), as modified by Yin *et al.* (2011), was used to estimate *g*_bs_ (see Supplementary appendix A at *JXB* online). The first modification was to include all four combinations of rate limitations to describe CO_2_ and light response curves of the C_4_ leaf CO_2_ assimilation rate (*A*) more smoothly:

$$A=\text{min}\left(A_{\text{EE}},A_{\text{ET}},A_{\text{TE}},A_{TT}\right)$$ (1)

where *A*_EE_ is the net CO_2_ assimilation rate when both C_4_ and C_3_ cycles are limited by enzyme activity, *A*_ET_ is the net rate when the C_4_ cycle is limited by enzyme activity and the C_3_ cycle is limited by e^−^ transport, *A*_TE_ is the rate when the C_4_ cycle is limited by e^-^ transport and the C_3_ cycle is limited by enzyme activity, and *A*_TT_ is the rate when both C_4_ and C_3_ cycles are limited by e^−^ transport. The formulation of von Caemmerer and Furbank (1999) used only two combinations, namely *A*=min(*A*_EE_,*A*_TT_). The second modification was to consider mesophyll conductance (*g*_m_) explicitly so that *A* is modelled using *C*_i_ (rather than mesophyll CO_2_ level *C*_m_) as input, as *C*_m_ is not measured. The model considering *g*_m_ becomes more complicated, and Yin *et al.* (2011) presented an analytical solution for each of the four limitations (see Supplementary appendix A). The third modification was to use the potential rate of ATP production (*J*_atp_), instead of the potential rate of e^−^ transport rate (*J*), because energy is partitioned between C_4_ and C_3_ cycles ultimately in terms of ATP (rather than in terms of electron) requirement. von Caemmerer and Furbank (1999) implicitly assumed that *J*_atp_=*J*, whereas the analysis of Yin and Struik (2012) showed that *J*_atp_ may not equal *J*.

We estimated the value of *J*_atp_ empirically from chlorophyll fluorescence measurements, according to Yin *et al.* (2011):

$$J_{\text{atp}}=s\prime I_{\text{inc}}(\Delta F/F_{\text{m}}\prime )/\left(\text{1}-x\right)$$ (2)

where *x* is the fraction of ATP partitioned to the C_4_ cycle (set to 0.4; von Caemmerer and Furbank 1999), and *s*′ is a lumped parameter resulting as the slope from the linear regression of *A* measured under low irradiances (≤500 µmol m^−2^ s^−1^) against *I*_inc_(∆*F*/*F*_m_′)/3 using only the data at 2% O_2_ combined with high CO_2_, at which photorespiration is suppressed. The intercept of the same linear regression will give the estimate of day respiration (*R*_d_) (Yin *et al.*, 2011). It is worth noting the importance of only using an e^−^ transport-limited range of data for estimating *s*′ which calibrates for the impact of any basal alternative e^−^ transport. Impacts of any additional alternative e^−^ transport, such as under high *I*_inc_ or low *C*_i_ conditions, arising from the higher *J*_atp_ than required for C_4_ and C_3_ cycles, are accounted for by Equation 1 via assigning to enzyme activity-limited rates.

The model of von Caemmerer and Furbank (1999) was proposed for the reference temperature 25 °C. To be applied for a range of varying temperatures, the potential variation of relative O_2_/CO_2_ diffusivities and solubilities with temperature needs to be quantified. This is presented in Supplementary appendix B, based on data in the literature (e.g. Frank *et al.*, 1996; Han and Bartels, 1996).

### Pre-determination of some photosynthetic parameters

To use the model to estimate *g*_bs_, a number of other input parameter values are required (see Table 1 for parameter definitions). The Rubisco kinetic parameters (*V*_cmax_, γ*, *K*_mC_, and *K*_mO_), the PEPc Michaelis–Menten constant (*K*_p_), and *R*_d_ are expected to increase with temperature, and this is conventionally described by an Arrhenius function normalized at 25 °C:

**Table 1.** Model input parameters, with their default values as derived from the literature or in this study

Symbol	Definition	Value	Source
*α*	Fraction of PSII activity in the bundle sheath	0.1	Chapman *et al.* (1980)
*R*_m_	Mitochondrial respiration in the mesophyll	0.5*R*_d_	von Caemmerer and Furbank (1999)
*x*	Fraction of ATP allocated to the C_4_ cycle	0.4	von Caemmerer and Furbank (1999)
*K*_p25_	Michaelis–Menten constant of PEPc for CO_2_ at 25 °C	40 μbar	Leegood and von Caemmerer (1989); Pfeffer and Peisker (1995)
*K*_mC25_	Michaelis–Menten constant of Rubisco for CO_2_ at 25 °C	485 μbar	Cousins *et al.* (2010)
*K*_mO25_	Michaelis–Menten constant of Rubisco for O_2_ at 25 °C	146 000 μbar	Cousins *et al.* (2010)
γ*_25_	Half the reciprocal of Rubisco specificity at 25 °C	0.0001747	Cousins *et al.* (2010)
*E*_Vcmax_	Activation energy for *V*_cmax_ (maximum Rubisco activity)	53.4 kJ mol^−1^	Sage (2002); Kubien *et al.* (2003); Perdomo *et al.* (2015)
*E*_γ*_	Activation energy for γ*	27.4 kJ mol^−1^	Derived from data of Jordan and Ogren (1984)
*E*_KmC_	Activation energy for *K*_mC_	35.6 kJ mol^−1^	Perdomo *et al.* (2015)
*E*_KmO_	Activation energy for *K*_mO_	15.1 kJ mol^−1^	Derived from results of Perdomo *et al.* (2015)
*E*_Vpmax_	Activation energy for *V*_pmax_ (maximum PEPc activity)	37.0 kJ mol^−1^	Derived from data of Chinthapalli *et al.* (2003)
*D*_Vpmax_	Deactivation energy for *V*_pmax_	214.5 kJ mol^−1^	Derived from data of Chinthapalli *et al.* (2003)
*S*_Vpmax_	Entropy term for *V*_pmax_	0.663 kJ K^−1^ mol^−1^	Derived from data of Chinthapalli *et al.* (2003)
*E*_Kp_	Activation energy for *K*_p_	68.1 kJ mol^−1^	Estimated in our report (see text)
*E*_gm_	Activation energy for *g*_m_ (mesophyll conductance)	49.6 kJ mol^−1^	Bernacchi *et al.* (2002)
*D*_gm_	Deactivation energy for *g*_m_	437.4 kJ mol^−1^	Bernacchi *et al.* (2002)
*S*_gm_	Entropy term for *g*_m_	1.4 kJ K^−1^ mol^−1^	Bernacchi *et al.* (2002)

PEPc, phospho*enol*pyruvate carboxylase; PSII, photosystem II.

$$\text{Parameter }={\text{Parameter}}_{25}\cdot e^{\frac{E}{R}\left(\frac{1}{298}-\frac{1}{273+T}\right)}$$ (3)

where *R* is the universal gas constant (0.008314 kJ K^−1^ mol^−1^) and *E* is the activation energy (kJ mol^−1^) for the parameter. It is impossible to derive a complete set of *in vivo* kinetic constants of C_4_ photosynthesis, and we mostly used *in vitro* values as reported in the literature. Values of γ*, *K*_mC_, *K*_mO_, and *K*_p_ at the reference temperature 25 °C (Table 1) were the same as those we used previously (Yin *et al.*, 2011), based on data for maize (e.g. Cousins *et al.*, 2010). Other parameter values are hardly available for maize, and the values used are summarized below.

(i) Sage (2002), Kubien *et al.* (2003), and Perdomo *et al.* (2015) reported the activation energy *E* of *V*_cmax_. Sage′s data on *E*_Vcmax_ for seven C_4_ species range from 50.1 kJ mol^−1^ for *Cynodon dactylon* and 53.5 kJ mol^−1^ for *Flaveria trinervia* to 68.0 kJ mol^−1^ for *Amaranthus retroflexus*. Kubien *et al*. (2013) reported 56.1 kJ mol^−1^ within 18–42 °C for *Flaveria bidentis*. *E*_Vcmax_ values reported by Perdomo *et al.* (2015) for *Flaveria bidentis* and *Flaveria trinervia* were 47.6 kJ mol^−1^ and 48.8 kJ mol^−1^, respectively. Despite the variation of *E*_Vcmax_ even for the same species, *E*_Vcmax_ for C_4_ species did not differ greatly from that for C_3_ species (Sage, 2002; Perdomo *et al.*, 2015). The average *E*_Vcmax_ of the three reports for C_4_ species was used here (Table 1).
(ii) Jordan and Ogren (1984) were the first to report on Rubisco specificity from 5 °C to 35 °C of a C_4_ species *Amaranthus hybridus*, from which we derived the activation energy for γ* (Table 1). This estimate is quite similar to the value of Bernacchi *et al.* (2002) for C_3_ species, and the report of Boyd *et al.* (2015) for a C_4_ species *Setaria viridis*.
(iii) The activation energy for *K*_mC_ of C_4_ Rubisco was based on the recent report of Perdomo *et al.* (2015) on two *Flaveria* C_4_ species (Table 1).
(iv) Little is known for the activation energy for *K*_mO_ of C_4_ Rubisco, and Table 1 gives its value that we derived from data of Perdomo *et al.* (2015) on activation energies for specificity (*S*_c/o_) and *K*_mC_, using the formula *K*_mO_=*S*_c/o_ *K*_mC_(*V*_omax_/*V*_cmax_) (where *V*_omax_ is the maximum oxygenation rate of Rubisco) and assuming that the *V*_omax_:*V*_cmax_ ratio does not vary with temperature (i.e. activation energy for this ratio=0). The latter assumption was based on reports that the activation energy for *V*_omax_:*V*_cmax_ of C_3_ Rubisco is either small (Bernacchi *et al.*, 2001) or inconsistent (either positive or negative) across species (von Caemmerer and Quick, 2000; Walker *et al.*, 2013). This is in line with the C_3_ photosynthesis modelling (Farquhar *et al.*, 1980) that *V*_omax_:*V*_cmax_ is set to be independent of temperature. The derived activation energy for *K*_mO_ (15.1 kJ mol^−1^) is only slightly higher than 10.5 (±4.8) kJ mol^−1^, the value that Boyd *et al.* (2015) published for *S. viridis* while we were revising our paper. Our *E*_KmO_ corresponds to a *Q*_10_ factor of ~1.23, very close to the value 1.20 that Chen *et al.* (1994) used. Sensitivity analysis showed that the temperature response of *g*_bs_ was least sensitive to *E*_KmO_ (see the Results).
(v) *V*_pmax_ follows an optimum response to temperature (Chinthapalli *et al.*, 2003; Massad *et al.*, 2007; Boyd *et al.*, 2015). This optimum response can be described by the peaked Arrhenius function (Medlyn *et al.* 2002):
  $$\text{Parameter }={\text{Parameter}}_{25}e^{\frac{E}{R}\left(\frac{1}{298}-\frac{1}{273+T}\right)}\frac{1+e^{\left(S-D/298\right)/R}}{1+e^{[S-D/(273+T)]/R}}$$ (4)
  where *S* is an entropy term (kJ K^−1^ mol^−1^), and *E* and *D* are energies of activation and deactivation (kJ mol^−1^), respectively. Differentiating Equation 4 gives the optimum temperature *T*_opt_ (°C) as:
  $$T_{\text{opt}}=\frac{D}{S+R\ln(D/E-1)}-273$$ (5)
We used the *in vitro* data of Chinthapalli *et al.* (2003) for *Amaranthus hypochondriacus*, which cover a very wide range of temperatures from 15 °C to 50 °C, to fit Equation 4 to derive values for *S*, *E*, and *D* of *V*_pmax_ (Table 1). These estimates resulted in an estimate of *T*_opt_=44.4 °C.
(vi) Little is known about the activation energy of *K*_p_. We examined the initial slope of *A*–*C*_i_ curves at 2% O_2_, since *A* at low *C*_i_ is limited by the PEPc activity (Sage and Kubien, 2007) and can be approximated to *C*_i_ *V*_pmax_/(*C*_i_+*K*_p_)–*R*_m_ (von Caemmerer and Furbank, 1999). The first-order derivative of this equation, d*A*/d*C*_i_, is *K*_p_ *V*_pmax_/(*C*_i_+*K*_p_)^2^, and was set to equal the slope value of the initial linear part of the *A*–*C*_i_ curve. The initial linear slope of the *A*–*C*_i_ curve followed an optimum response to temperature (see the Results) and this response is expected to result from temperature responses of both *K*_p_ and *V*_pmax_. Using the pre-estimated temperature response parameters of *V*_pmax_, we then derived the activation energy for *K*_p_ by fitting combined Equation 3 and d*A*/d*C*_i_=*K*_p_ *V*_pmax_/(*C*_i_+*K*_p_)^2^ to data on the initial linear slope of the *A*–*C*_i_ curves over the six temperatures. We will confirm our estimate on *E*_Kp_ from fitting a full model to data of the initial part of *A*–*C*_i_ curves (see the Results).
(vii) Mesophyll conductance (*g*_m_) may be a significant limiting factor for C_4_ photosynthesis (Pfeffer and Peisker, 1998), has an impact on estimation of leakiness (von Caemmerer *et al.*, 2014), and its role in estimating *g*_bs_ has yet to be quantified. However, *g*_m_ for C_4_ photosynthesis is hard to estimate (Pfeffer and Peisker, 1998; Barbour *et al.*, 2016), let alone its temperature response parameters. We took the widely used values of Bernacchi *et al.* (2002) for *g*_m_ in C_3_ photosynthesis, which include *E*, *D*, and *S* as quantified in Equation 4 (Table 1). This approach assumes that C_3_ and C_4_ photosynthesis have a similar relative response of *g*_m_ to temperature, although *g*_m_ in C_4_ does not involve chloroplast-related resistance components. Our assumption for the same relative response of the overall *g*_m_ to temperature for C_3_ and C_4_ leaves will be tested through a sensitivity analysis.

### Curve fitting and sensitivity analysis

With all these parameters pre-determined, we estimated *g*_bs_ of the six temperatures as well as *g*_m25_, *V*_cmax25_, *V*_pmax25_ (i.e. *g*_m_, *V*_cmax_, *V*_pmax_ at 25 °C, respectively), by the non-linear curve-fitting using Equation 1 in combination with solutions in Supplementary appendix A. We used a dummy variable approach (Yin *et al.*, 2009), in which we introduced six dummy variables to correspond to six temperatures, allowing us to estimate treatment-specific parameters (i.e. *g*_bs_ at six temperatures) and common parameters (i.e. *g*_m25_, *V*_cmax25_, and *V*_pmax25_) simultaneously. The statistical fitting algorithms, implemented in SAS, autoassigned the range of data points to each of the four limitations as defined by Equation 1. The *g*_bs_ estimates when plotted against leaf temperature followed an optimum response (see the Results), and parameters characterizing this response were derived from fitting the estimated *g*_bs_ to Equation 4. All required curve fitting was carried out using the least-squares non-linear regression with the GAUSS method in PROC NLIN of SAS (SAS Institute Inc., Cary, NC, USA). The SAS codes for estimating *g*_bs_ parameters are available from the corresponding author upon request.

Regardless of the technique used, estimates for kinetic constants of Rubisco are full of uncertainties (Kubien *et al*., 2008; Cousins *et al.*, 2010), so are the constants of PEPc (Pfeffer and Peisker, 1998) and of *g*_m_ (Silim *et al.*, 2010; Walker *et al.*, 2013). Also, the input values of some constants were not determined exclusively for maize. Therefore, a full sensitivity analysis on the *g*_bs_ estimates was conducted with respect to these input parameters in Table 1, except for *x* and α. The value of *x* is expected to be very invariant in terms of the ATP requirement between C_4_ and C_3_ cycles (von Caemmerer and Furbank, 1999), and a previous analysis (Yin *et al.*, 2011) showed little sensitivity of *g*_bs_ to parameter α.

## Results

### Overall response curves of A and ∆*F*/*F*_m_′ to CO_2_ and irradiance

**Fig. 1.**
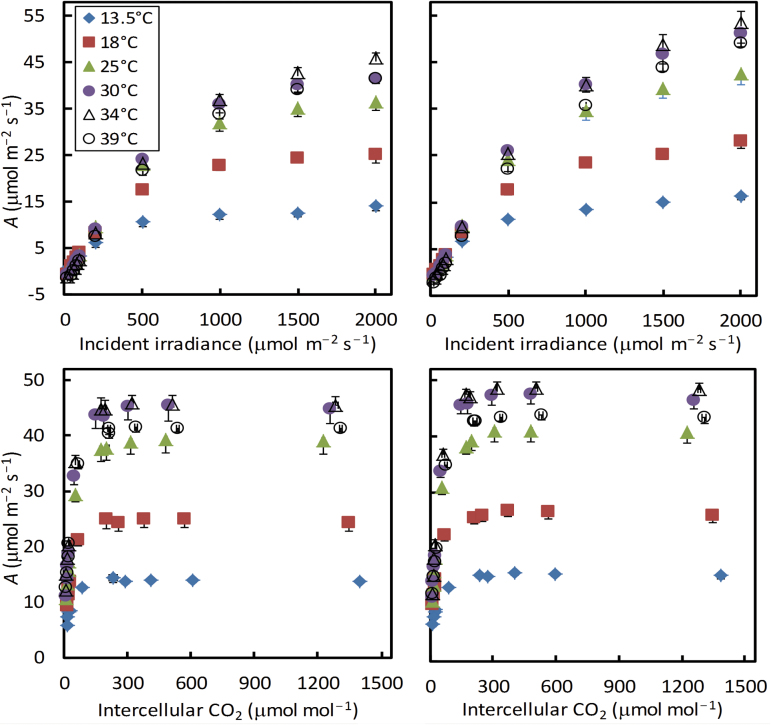
Net CO_2_ assimilation rate *A* at six leaf temperatures in response to incident irradiance or to intercellular CO_2_ levels under 21% (left panels) or 2% (right panels) O_2_ conditions. Each symbol represents the mean of four replicated leaves (SEMs are visible in bars if larger than the symbols).(This figure is available in colour at *JXB* online.)

**Fig. 2.**
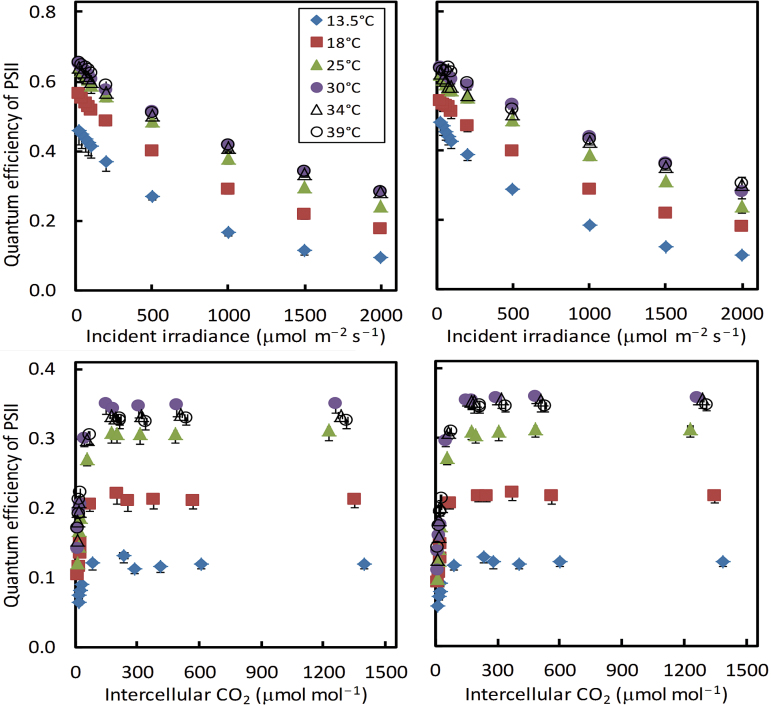
Apparent operating quantum efficiency of photosystem II (PSII) electron transport (Φ_2_ or ∆*F*/*F*_m_′) at six leaf temperatures in response to incident irradiance or to intercellular CO_2_ levels under 21% (left panels) or 2% (right panels) O_2_ conditions. Each symbol represents the mean of four replicated leaves (SEMs are visible if larger than the symbols). (This figure is available in colour at *JXB* online.)

Our experimental results at the six temperatures showing typical irradiance and CO_2_ responses of the C_4_ photosynthesis rate were obtained (Fig. 1). The non-photorespiratory condition (2% O_2_ combined with 1000 μmol mol^−1^ *C*_a_) had a moderate positive effect on the irradiance response curves, compared with the curves of 21% O_2_ combined with 250 μmol mol^−1^ *C*_a_. Temperature strongly affected both irradiance and CO_2_ response curves, and its effect was more significant from 13.5 °C to 25 °C than from 25 °C to 39 °C. The maximum photosynthesis was observed at ~34 °C. The effect of temperature on photosynthesis was reflected by the data for temperature effect on ∆*F*/*F*_m_′, the apparent operating efficiency of PSII e^−^ transport (Fig. 2).

### Estimates of *s′* and *R*_d_

**Fig. 3.**
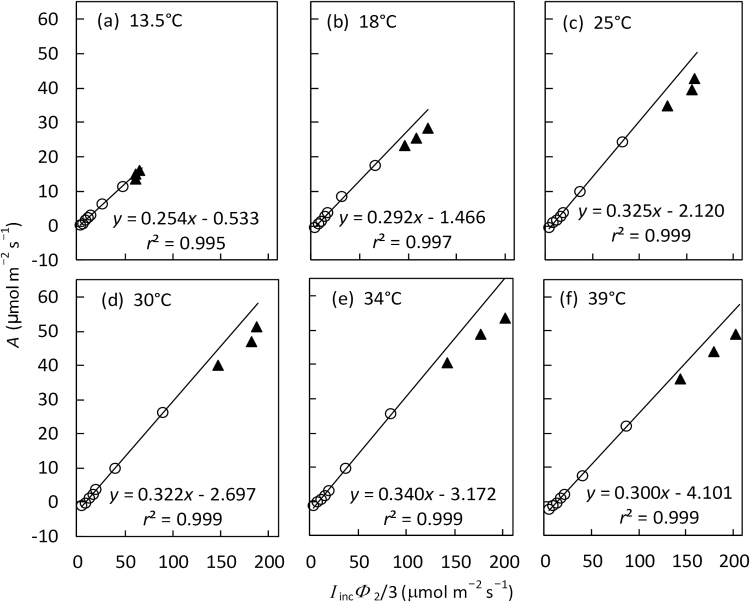
The relationship between net CO_2_ assimilation rate *A* and the lumped variable *I*_inc_Φ_2_/3 (mean of four replicates) from irradiance response curves under non-photorespiratory conditions (i.e. at 2% O_2_ combined with high CO_2_) at six leaf temperatures. Open circles are for *I*_inc_ ≤500 μmol m^−2^ s^−1^ and filled triangles come from the three levels of *I*_inc_ >500 μmol m^−2^ s^−1^. The lines represent linear regression based on data with *I*_inc_ ≤500 μmol m^−2^ s^−1^, in which the slope gives the estimate of calibration factor *s*′ and the intercept gives the estimate of day respiration *R*_d_ (see the text).

The relationship between *A* and *I*_inc_(∆*F*/*F*_m_′)/3 measured at low irradiances (≤500 µmol m^−2^ s^−1^) under non-photorespiratory conditions was linear for all temperatures (Fig. 3). The slope of this linear relationship gives the estimate for *s*′, a lumped parameter for calculating *J*_atp_ (see Equation 2), and its intercept gives the estimate of *R*_d_ (day respiration). Data points of higher irradiances (>500 µmol m^−2^ s^−1^) lay below the linear trend for all temperatures (Fig. 3), indicating that *A* was limited by *A*_EE_ or *A*_TE_ (see Equation 1) at high irradiances.

**Fig. 4.**
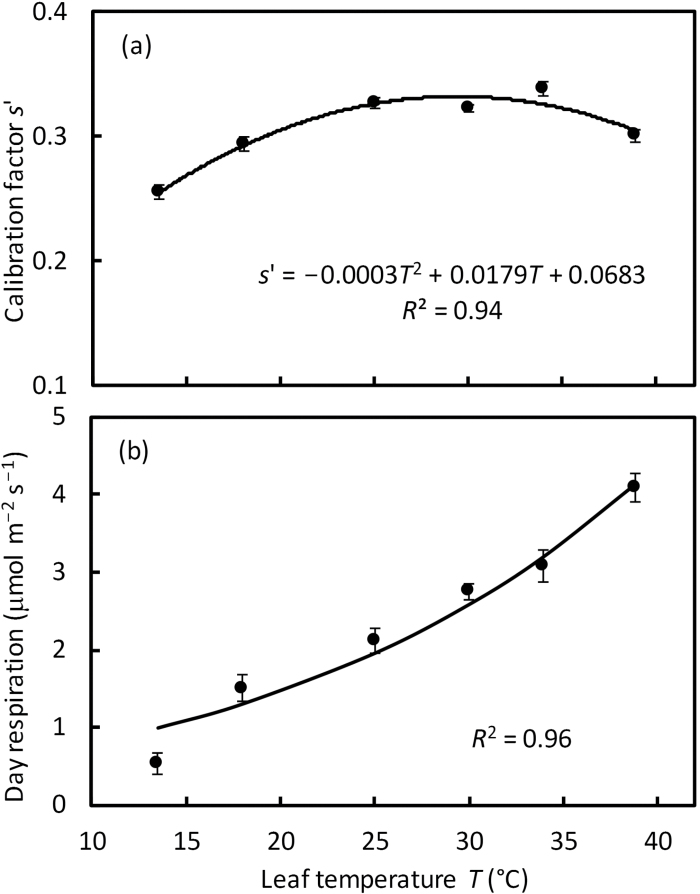
(a) Temperature response of the estimated calibration factor *s*′, and (b) temperature response of the estimated day respiration *R*_d_. Values of *s*′ and *R*_d_ were estimated as the slope and the intercept, respectively, of linear regression in Fig. 3. In (b), the curve represents the Arrhenius plot of Equation 3 with estimated parameter values in Table 2. Bars in (a) and (b) represent SEs of the estimates.

The estimated calibration factor *s*′ varied from 0.25 to 0.34, and its response to temperature can be empirically described by a quadratic equation with the optimum temperature at ~30 °C (Fig. 4a). As expected, the estimated values of *R*_d_ increased with increasing temperature (Fig. 4b). This response was well described by the Arrhenius equation, Equation 3, with the estimated *R*_d_ at 25 °C being 1.95 μmol m^−2^ s^−1^ and the activation energy being 41.9 kJ mol^−1^ (Table 2).

**Table 2.** Values (standard errors of the estimates in parentheses) of parameters at the reference temperature 25 °C, activation energy *E* in Equation 3 for day respiration (*R*_d_), as well as activation energy *E*, deactivation energy *D*, and entropy term *S* of Equation 4 for bundle-sheath conductance (*g*_bs_) and its optimum temperature *T*_opt_ calculated from Equation 5, of maize leaves, as estimated from data in the present study

Parameter	Estimate at 25 °C	*E* (kJ mol^−1^)	*D* (kJ mol^−1^)	*S* (kJ K^−1^ mol^−1^)	*T*_opt_ (°C)
*R*_d_	1.95(0.14) μmol m^−2^ s^−1^	41.85 (5.32)	NA	NA	NA
*g*_m_	1.33(0.06) mol m^−2^ s^−1^	–	–	–	–
*V*_cmax_	49.0(0.9)μmol m^−2^ s^−1^	–	NA	NA	NA
*V*_pmax_	119.2(4.1)μmol m^−2^ s^−1^	–	–	–	–
*g*_bs_	2.87(0.31) mmol m^−2^ s^−1^	116.77 (30.39)	264.60 (51.96)	0.86 (0.16)	33.9

NA, not applicable; –, not estimated from data of the present study, and most of them are given in Table 1, based on data in the literature, and were used as input to our present model analysis.

### Initial slope of *A–* *C*_i_ curves

**Fig. 5.**
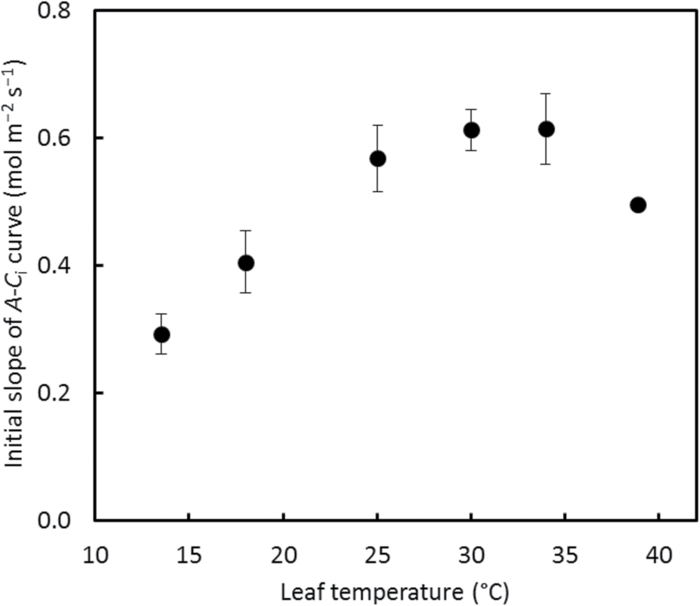
Temperature response of the initial slope of the *A*–*C*_i_ curve at 2% O_2_. The error bar of each point indicates ±SEM of four replicated leaves. The error bar of the last point is smaller than the symbol.

The *A*–*C*_i_ curves at 2% O_2_ for the first three (for 13.5 °C) or four (for the remaining temperatures) points were essentially linear (Supplementary Fig. S1). The slope of this initial linear section of *A*–*C*_i_ curves at six temperatures followed an optimum response to temperature (Fig. 5), and this response is expected to result from temperature responses of both *K*_p_ and *V*_pmax_. Using the pre-estimated temperature response parameter values of *V*_pmax_ (Table 1), we estimated the activation energy for *K*_p_ by fitting combined Equation 3 and d*A*/d*C*_i_=*K*_p_ *V*_pmax_/(*C*_i_+*K*_p_)^2^ to data in Fig. 5 for the initial slope of *A*–*C*_i_ curves at the six temperatures. The estimated *E*_Kp_, the activation energy for *K*_p_, was 68.1 (SE 6.8) kJ mol^−1^ (Table 1).

### Estimated response of *g*_bs_ to temperature

With *s*′, *R*_d_, and *E*_Kp_ pre-fixed as presented above and other input parameters available (Table 1), we were able to estimate *g*_m25_, *V*_cmax25_, *V*_pmax25_, and *g*_bs_ of the six temperatures by fitting our model (Equation 1 combined with solutions given in the Supplementary data) to all data collected in the experiment. The model described the whole data set across *A*–*C*_i_ and *A*–*I*_inc_ curves at 2% and 21% O_2_, with *R* ^2^=0.98 and relative root-mean-square error rRMSE (RMSE×100/the mean of measured *A*)=12.0%, and a plot comparing modelled and measured *A*–*I*_inc_ and *A*–*C*_i_ curves is given in Supplementary Fig. S2 for 21% O_2_. The obtained *g*_m25_ was 1.33mol m^−2^ s^−1^, *V*_cmax25_ was 49.0 μmol m^−2^ s^−1^, and *V*_pmax25_ was 119.2 μmol m^−2^ s^−1^ (Table 2), meaning that the *V*_pmax25_:*V*_cmax25_ ratio is 2.43. We measured leaf N content, which was 1.10 (SE 0.05) g m^−2^. Assuming the base leaf nitrogen content for photosynthesis is 0.24g m^−2^ (Yin *et al.* 2011), the slope of *V*_cmax_ and *V*_pmax_ versus leaf N is 57.7 μmol and 140.3 μmol (g N)^−1^ s^−1^, respectively.

**Fig. 6.**
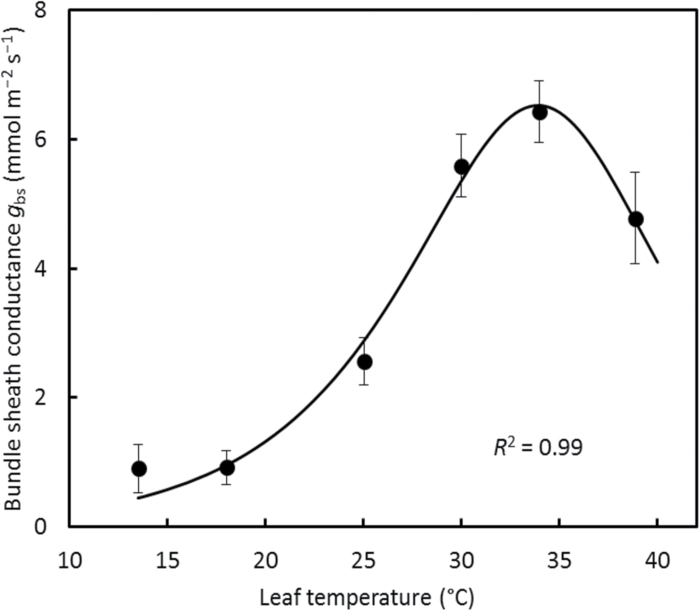
Temperature response of estimated bundle-sheath conductance *g*_bs_ in maize leaves. The error bar at each point represents ±SE of the estimate. The curve represents the peaked Arrhenius fit of Equation 4 with estimated values of the parameters in Table 2.

The estimated *g*_bs_ clearly varied with leaf temperature, and this response can be well described by the peaked Arrhenius equation, Equation 4 (Fig. 6). The obtained parameters of Equation 4 are: *g*_bs25_=2.87 mmol m^−2^ s^−1^, *E*=116.7 kJ mol^−1^, *D*=264.6 kJ mol^−1^, and *S*=0.86 kJ K^−1^ mol^−1^ (Table 2). These parameter values of the peaked Arrhenius equation predicted, according to Equation 5, 34 °C as the optimum temperature for *g*_bs_.

If we set *g*_bs_ independent of temperature, the obtained estimates from fitting to our data would be: *g*_m25_=4.37mol m^−2^ s^−1^, *g*_bs_=4.53 mmol m^−2^ s^−1^, *V*_cmax25_=52.7 μmol m^−2^ s^−1^, and *V*_pmax25_=80.0 μmol m^−2^ s^−1^, with *R* ^2^=0.98 and rRMSE=13.3%. The obtained *g*_m25_ increased by >3-fold, *V*_pmax25_ decreased by 33%, and, therefore, the *V*_pmax25_:*V*_cmax25_ ratio dropped to 1.5 and may have been underestimated (see the Discussion). Furthermore, this *g*_bs_ temperature-insensitive model statistically decreased the goodness of fit (*P*<0.001 based on the *F*-test).

If we use the original model of von Caemmerer and Furbank (1999) which predicts *A* as the minimum of two limiting *A*_TT_ and *A*_EE_, we obtained the estimates: *g*_m25_=1.53mol m^−2^ s^−1^, *V*_cmax25_=41.9 μmol m^−2^ s^−1^, and *V*_pmax25_=119.8 μmol m^−2^ s^−1^, with *g*_bs_ being 1.18, 0.72, 3.39, 10.60, 9.35, and 5.07 mmol m^−2^ s^−1^ at 13.5, 18, 25, 30, 34, and 39 °C, respectively. The model described the data (*R* ^2^=0.97 and rRMSE=14.3%) somewhat less adequately than our four-rates model. Also, the estimates of *g*_bs_ at 30 °C and 34 °C became higher, resulting in different parameter estimates for *g*_bs_ temperature response: *g*_bs25_=3.54 mmol m^−2^ s^−1^, *E*=264.9 kJ mol^−1^, *D*=385.7 kJ mol^−1^, and *S*=1.27 kJ K^−1^ mol^−1^. This gives *T*_opt_=31.3 °C, ~2.6 °C lower than *T*_opt_ resulting from the four-rates model. The difference stemmed from the fact that many data points were determined by *A*_TE_ (results not shown), which is excluded in the two-rates model.

### Sensitivity of estimated *g*_bs_–temperature relationships to input parameters

In Table 1, the value of *E*_Kp_ was derived from our own data using an approximate model for describing the initial slope of *A*–*C*_i_ curves (see earlier). This procedure may be criticized because (i) the approximate model assumes an infinite *g*_m_; (ii) the procedure requires that the initial section of *A*–*C*_i_ curves is exactly linear; and (iii) the required estimates for temperature response parameters of *V*_pmax_, which we derived from Chinthapalli *et al.* (2003), may actually be uncertain. To examine the uncertainties in our estimated *E*_Kp_, we used a more complete model (Supplementary appendix C) combined with two other reports (Massad *et al.*, 2007; Boyd *et al.*, 2015) on temperature response parameters of *V*_pmax_. We fitted the model to the initial section of *A–C*_i_ curves where *A* is expected to be limited by the PEPc activity, and the obtained *E*_Kp_ estimate was 66.3, 79.5, and 73.3 kJ mol^−1^ if temperature response parameters of *V*_pmax_ came from Chinthapalli *et al.* (2003), Massad *et al.* (2007), and Boyd *et al.* (2015), respectively (Supplementary Table S1). When the *E*_Kp_ estimate was combined with their corresponding temperature response parameter values of *V*_pmax_ from the three studies, the resulting estimates of *g*_bs_ at six temperatures and of *V*_cmax25_ were hardly affected by the use of these different sets of input for *E*_Kp_ and temperature response of *V*_pmax_ (Supplementary Table S1).

The temperature responses of *g*_m_ do not yet exist for C_4_ species, and temperature responses for C_3_ species differed greatly among reports for tobacco (Bernacchi *et al.*, 2002; Evans and von Caemmerer, 2013; Walker *et al.*, 2013), and among species (von Caemmerer and Evans, 2015), in particular between tobacco and Arabidopsis (Walker *et al.*, 2013). The responses for tobacco ranged from the peaked Arrhenius response (Bernacchi *et al.*, 2002; Walker *et al.*, 2013) to a linear pattern (Evans and von Caemmerer, 2013), whereas data of Walker *et al.* (2013) for Arabidopsis showed virtually no effect of temperature on *g*_m_. Despite such a contrast in the temperature response of *g*_m_ used as input, the overall response of *g*_bs_ to temperature remained similar, following a peaked Arrhenius pattern (Supplementary Fig. S3). The obtained *V*_cmax25_ varied little, from 48.5 μmol m^−2^ s^−1^ to 51.4 μmol m^−2^ s^−1^. The obtained *V*_pmax25_ varied to a greater extent, from 75.3 μmol m^−2^ s^−1^ (from using the Arabidopsis response) to 155.6 μmol m^−2^ s^−1^ (from using the tobacco response of Walker *et al.*, 2013). The obtained *g*_m25_ was mostly between 1.25mol m^−2^ s^−1^ and 1.45mol m^−2^ s^−1^, but using the Arabidopsis response gave an infinite estimate of *g*_m25_.

**Fig. 7.**
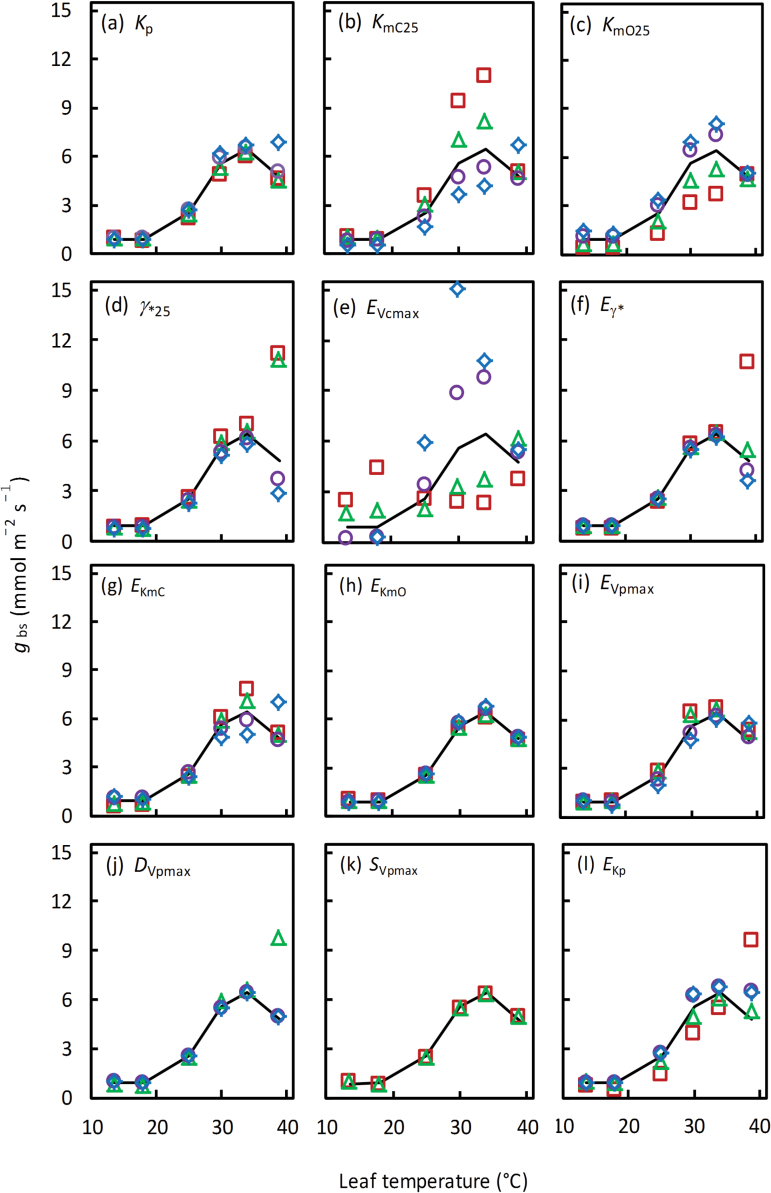
Sensitivity of bundle-sheath conductance, *g*_bs_, temperature response to changes in 12 input parameters as shown in (a–l). The input parameters and their default values are defined in Table 1. The changes were made to be 0.50 (open squares), 0.75 (open triangles), 1.25 (open circles), and 1.50 (open diamonds) times their default value. The temperature response of *g*_bs_ using the default set of input parameter values is given by the solid curve of each panel. One or two types of symbols are missing in (j) and (k) because extreme values of either *D*_Vpmax_ or *S*_Vpmax_ resulted in a biologically unrealistic negative estimate of *g*_bs_. (This figure is available in colour at *JXB* online.)

For sensitivity analyses of the estimated *g*_bs_ temperature response to 12 other parameters, the input parameters were varied by ±25% and 50% of their default values (Fig. 7). The estimated temperature response of *g*_bs_ was least sensitive to changes in *K*_p_ (Fig. 7a), *E*_KmO_ (Fig. 7h), and *E*_Vpmax_ (Fig. 7i). Overall, the sensitivity depended on the level of the parameter changes. Using extreme values of γ*_25_, *E*_Vcmax_, *E*_γ*_, *E*_KmC_, *D*_Vpmax_, and *E*_Kp_ changed the shape of the response, from the peaked optimum response to the non-peaked Arrhenius pattern. The −50% change in *D*_Vpmax_ and +25% and +50% changes in *S*_Vpmax_ resulted in a biologically unrealistic (negative) estimate of *g*_bs_, so their resulting response pattern is not given (Fig. 7j, k), suggesting that these changes may have reached beyond biologically realistic scopes of the two parameters. Note that Equation 5 suggests a co-determination of *T*_opt_ by *E*, *D*, and *S* and a much higher sensitivity to *D*_Vpmax_ and *S*_Vpmax_ than to *E*_Vpmax_ in determining *T*_opt_ of *V*_pmax_.

The ±25% and 50% changes of the 12 parameters also resulted in changes in estimated *g*_m25_, *V*_cmax25_, and *V*_pmax25_ (results not shown). Overall, the estimated *V*_cmax25_ varied least (from 45.7 μmol m^−2^ s^−1^ to 58.4 μmol m^−2^ s^−1^), and its relative change, defined as the difference between its maximum and minimum divided by its mean, was 26%, whereas *V*_pmax_ varied most (from 67.6 μmol m^−2^ s^−1^ to 322.6 μmol m^−2^ s^−1^), and its relative change was 195%. The relative change of the estimated *g*_m25_ was 92% (from 0.93mol m^−2^ s^−1^ to 2.05mol m^−2^ s^−1^).

### Estimated leakiness, and *V*_o_:*V*_c_ and *V*_c_:*V*_p_ ratios

**Fig. 8.**
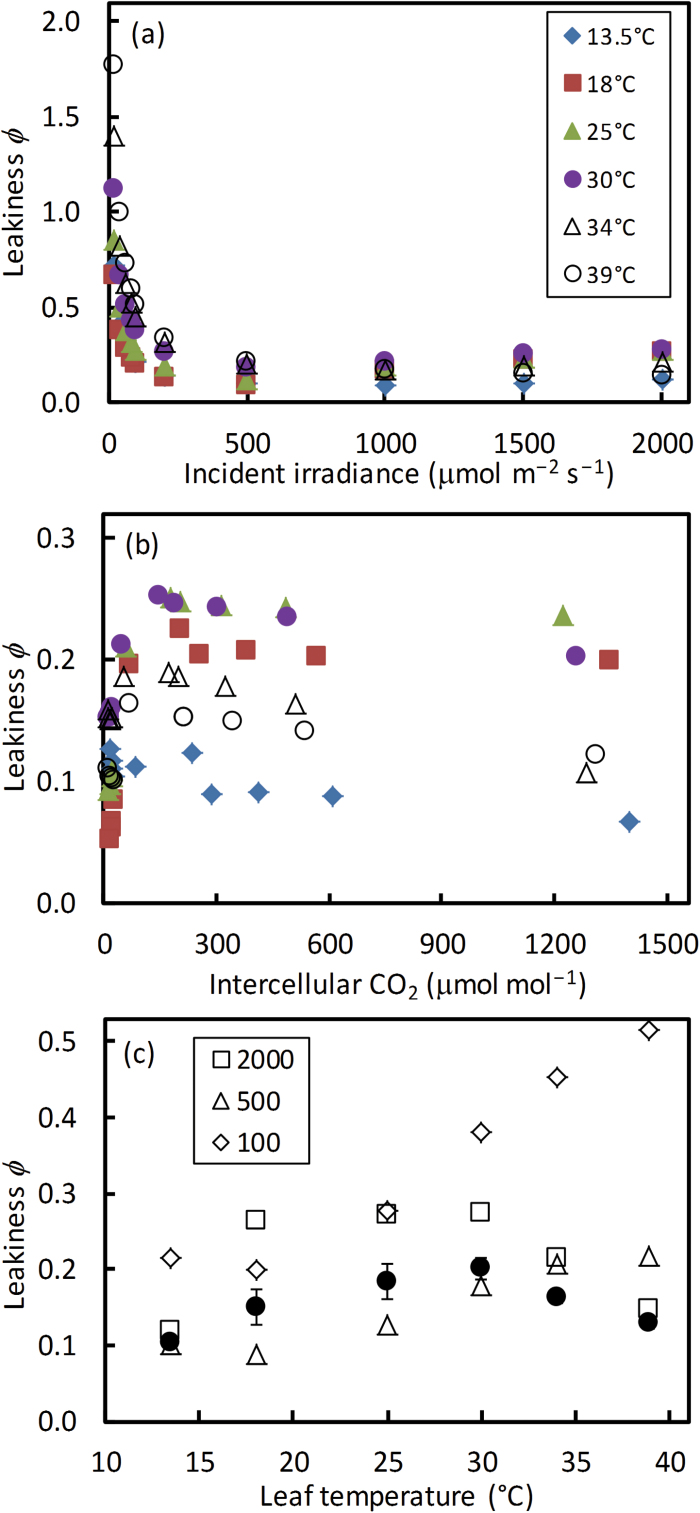
Calculated CO_2_ leakiness ϕ as a function of irradiance (a) and of intercellular CO_2_ level (b) at six temperatures. The values of leakiness ϕ from (a) for three contrasting irradiance levels of 100, 500, and 2000 μmol m^−2^ s^−1^ (open symbols) and the mean ϕ (SEM in bars) across all CO_2_ levels from (b) (filled circles) are shown as a function of temperature (c). The O_2_ level was at 21%.(This figure is available in colour at *JXB* online.)

When model parameters are estimated, one can solve for leakiness ϕ (*=L/V*_p_), *V*_o_:*V*_c_, and *V*_c_:*V*_p_ ratios, using Equations A1–A6 in Supplementary appendix A. The calculated ϕ declined sharply with increasing irradiance (Fig. 8a), and increased initially and then saturated with increasing *C*_i_ (Fig. 8b). At low irradiances, ϕ values were very high, even exceeding 1.0 when the temperature was >30 °C (Fig. 8a). The temperature response of ϕ did not vary with CO_2_ levels but depended strongly on the irradiance levels (Fig. 8c). The estimated ϕ at high irradiance (2000 μmol m^−2^ s^−1^) and the average ϕ of various CO_2_ levels showed a peaked response to temperature (Fig. 8c).

**Fig. 9:**
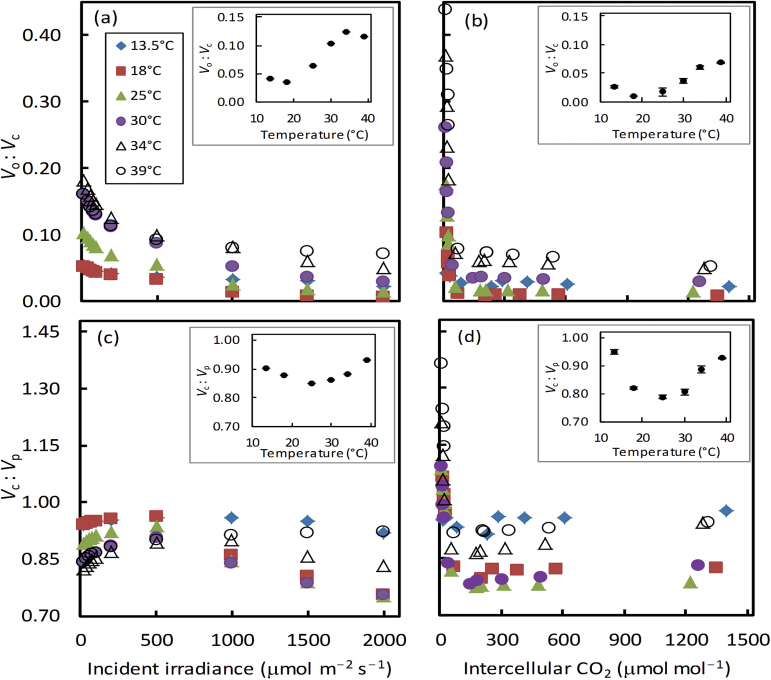
Calculated *V*_o_:*V*_c_ ratios (a, b) and *V*_c_:*V*_p_ ratios (c, d) as a function of irradiance (a, c) and of intercellular CO_2_ level (b, d) at six temperatures. The mean ratios (SEM in bars if larger than symbols) across all irradiance levels and across CO_2_ levels with *C*_a_ ≥200 μmol mol^−1^ against temperature during measurements are shown in the inset of each panel. The O_2_ level was 21%.(This figure is available in colour at *JXB* online.)

The calculated *V*_o_:*V*_c_ ratio varied slightly with irradiance (Fig. 9a), and initially declined sharply and then became stable with increasing CO_2_ levels (Fig. 9b). In accordance with this pattern, the *V*_c_:*V*_p_ ratio responded to irradiance and CO_2_ levels (Fig. 9c, d). The *V*_c_:*V*_p_ ratio was <1.0 across irradiances (Fig. 9c), and it was also <1.0 for most CO_2_ levels but became >1.0 at low *C*_i_ of 10–30 μmol mol^−1^, especially at high temperatures (Fig. 9d). Excluding the four low CO_2_ levels, the average ratios were calculated to show how these ratios under normal irradiance and CO_2_ conditions responded to temperature (inset in each panel of Fig. 9). Overall, the *V*_o_:*V*_c_ ratio increased with temperature (insets in Fig. 9a, b), and the *V*_c_:*V*_p_ ratio had a non-linear response to temperature (insets in Fig. 9c, d).

## Discussion

To estimate *g*_bs_, we used the model method of Yin *et al.* (2011), which is based on the combined measurements of gas exchange and chlorophyll fluorescence on leaves. There are some concerns about this technique as the distribution of e^−^ transport between mesophyll and bundle-sheath cells on C_4_ leaves is uncertain (von Caemmerer, 2013; Kromdijk *et al.*, 2014). The mesophyll and bundle-sheath cells have different chloroplast populations which could result in a complex relationship between ∆*F*/*F*_m_′ and the quantum yield of CO_2_ fixation. However, despite a few exceptions (e.g. Fryer *et al.*, 1998; Dwyer *et al.*, 2007), most studies (e.g. Krall and Edwards, 1990; Edwards and Baker, 1993; Oberhuber and Edwards, 1993; Oberhuber *et al.*, 1993; Peterson, 1994; Earl and Tollenaar, 1998; Laisk and Edwards, 1998; Siebke *et al.*, 2003; Naidu and Long, 2004; Loriaux *et al.*, 2013; Bellasio and Griffiths, 2014) have reported a good linear relationship for C_4_ species between (quantum yields of) PSII e^−^ transport from fluorescence analysis and CO_2_ fixation from gas exchange data over a wide range of conditions. This empirical evidence suggests that combined gas exchange and chlorophyll fluorescence measurements, commonly applied to estimate C_3_ photosynthesis parameters, can be similarly applied for C_4_ photosynthesis, as implemented by Yin *et al.* (2011), Bellasio and Griffiths (2013, 2014), and Bellasio *et al.* (2016). The way we conducted calibrations using non-photorespiratory measurements to derive *s*′ for calculating *J*_atp_ may also have reduced the uncertainty of applying a chlorophyll fluorescence technique to C_4_ leaves.

In fact, the estimate of *s*′ is not just a calibration factor, but has physiological meanings and integrates a number of hard to determine parameters (Yin *et al.*, 2011):

$$s'=\left(\text{1}-x\right)\beta \rho_{2}z\xi$$ (6)

where β is absorptance by leaf photosynthetic pigments, ρ_2_ is the fraction of absorbed irradiance partitioned to PSII, *z* is the factor of converting PSII e^−^ flux into ATP flux, and ξ is the ratio of true PSII efficiency to fluorescence-measured apparent PSII efficiency. Theoretically, ρ_2_ and *z* can be written as (Yin and Struik. 2012):

$$\rho {\text{​}}_{2}=\frac{1-f_{\text{cyc}}}{\left(1-f_{\text{cyc}}\right)+\Phi_{2\text{LL}}/\Phi_{1\text{LL}}}$$ (7)

$$z=\frac{2+f_{\text{Q}}-f_{\text{cyc}}}{h\left(1-f_{\text{cyc}}\right)}$$ (8)

where *f*_cyc_ is the fraction of e^−^ flux at PSI that follows cyclic transport, *f*_Q_ is the fraction of e^−^ flux at reduced plastoquinone that follows the Q cycle, *h* is the H^+^:ATP ratio, and Φ_2LL_/Φ_1LL_ is the PSII:PSI e^−^ transport efficiency ratio. Given recent quantitative estimation that Φ_2LL_/Φ_1LL_= ~0.825, *f*_Q_=1, *f*_cyc_= ~0.45, and *h*=4 (Yin and Struik, 2012), and assuming that *x*=0.4, β=0.9, and ξ=1, the value of *s*′ must be ~0.25. This theoretical value is close to our estimates for *s*′, 0.25–0.34 (Figs 3, 4a). Combining Equations 6–8 with the equation of Yin and Struik (2012) for the condition that the produced NADPH and ATP from e^−^ transport match the metabolic requirements (their equation 5), *s*′ can also be expressed as:

$$s'=\frac{3}{4}\beta \rho {\;}_{2}\xi (1+x\phi )\left(1-\frac{f_{\text{pseudo}}}{1-f_{\text{cyc}}}\right)$$ (9)

where ϕ is leakiness, *f*_pseudo_ is the fraction of e^−^ flux at PSI that follows the basal pseudocyclic transport (e.g. nitrate reduction, and malate export from chloroplasts), and the term [1−*f*_pseudo_/(1−*f*_cyc_)] as a whole refers to the fraction of the PSII e^−^ flux that is used for supporting the Calvin circle and any photorespiration (Yin and Struik, 2012). Therefore, our calibration factor *s*′ takes into account only the part of the fluorescence signal dedicated to e^−^ sinks represented by the Calvin cycle, any photorespiration, and some energy loss due to CO_2_ leakage. Our calibration procedure has excluded the effect of possible basal alternative e^−^ sinks. For example, the calibration factor *s*′ was found to vary with temperature (Fig. 4a), and one possible reason for this variation is that the extent of any basal alternative e^−^ transport may depend on temperature.

Kromdijk *et al.* (2010) and Ubierna *et al.* (2013) estimated *g*_bs_ by fitting the model of von Caemmerer and Furbank (1999) to carbon isotope discrimination data that were measured simultaneously with gas exchange, thereby providing an independent method to estimate *g*_bs_. As this isotopic method has more assumptions than the fluorescence-based method (Kromdijk *et al.*, 2014) and does not estimate *J*_atp_ and *R*_d_, Bellasio and Griffiths (2013) compared the two methods in estimating *g*_bs_ by using *J*_atp_ and *R*_d_ estimated along the lines of our method. It is noteworthy that the fluorescence method used by Bellasio and Griffiths (2013) slightly differs from our method in that they estimated *g*_bs_ by minimizing the difference between modelled and measured *J*_atp_, whereas our fitting method is to minimize the difference between modelled and measured *A*. Yin and Struik (2009*b*) have shown for C_3_ photosynthesis that the two minimizing targets can result in slightly different estimates of *g*_m_, and we prefer our method because it is generally *A*, rather than *J*_atp_, that is to be predicted from the general use of photosynthesis models. Nevertheless, Bellasio and Griffiths (2013) found that, compared with *g*_bs_ estimated by the isotopic method, *g*_bs_ estimated by the fluorescence method by fitting to *J*_atp_ was similar for maize leaves grown under low light conditions but was much lower for leaves from high light conditions; and the reasons for the difference are unresolved (Kromdijk *et al.*, 2014). Bellasio and Griffiths (2013) discussed several advantages of the fluorescence method compared with the isotopic method (e.g. lower noise/signal ratio). However, since the isotopic method does not have the same problem as the fluorescence method in dealing with the two cell types, it is necessary to study further whether the relative temperature response of *g*_bs_ we obtained here (Figs 6, 7) can be confirmed using the independent isotopic method.

Our model considered only two major enzymes (i.e. PEPc for the C_4_ cycle and Rubisco for the C_3_ cycle). Other enzymes [e.g. pyruvate orthophosphate dikinase, C_4_-acid decarboxylase, and carbonic anhydrase (CA)] are also important. The fact that detailed kinetic constants of these enzymes are rare and uncertain forces us to consider only two enzymes as in most applications of the C_4_ model of von Caemmerer and Furbank (1999). Even for these two enzymes, only *in vitro* estimates of kinetic constants were used here as their *in vivo* estimates are practically impossible to obtain, and, when estimated, possibly confounded by the assumptions made on other parameters. For example, Massad *et al.* (2007) assumed that *g*_bs_ and γ* are independent of temperature, and obtained *in vivo* estimates of the peaked Arrhenius temperature response (i.e. Equation 4) for *V*_cmax_ and *V*_pmax_, with *T*_opt_=32.5°C and 43.3 °C, respectively. Their *T*_opt_ estimate for *V*_pmax_ is similar to our estimate of 44.4 °C based on *in vitro* data of Chinthapalli *et al.* (2003). However, the peaked temperature response for *V*_cmax_ has seldom been observed *in vitro*, for both C_3_ and C_4_ Rubisco, even when the temperature is up to 35–40 °C (e.g. Badger and Collatz, 1977; Jordan and Ogren, 1984; Sage, 2002; Kubien *et al.*, 2003; Walker *et al.*, 2013; Boyd *et al.*, 2015; Perdomo *et al.*, 2015). We believe that the low *T*_opt_ for *V*_cmax_ obtained by Massad *et al.* (2007) could simply be because they ignored the temperature sensitivity of *g*_bs_ and γ* when estimating *V*_cmax_.

Our analysis showed the necessity of accounting for temperature response of *g*_bs_. The shape of our temperature response of *g*_bs_ using default parameters (Fig. 6) followed an optimum temperature response pattern of light- and CO_2_-saturated photosynthesis rates (*A*_max_), similar to that Bernacchi *et al.* (2002) and others obtained for *g*_m_ in C_3_ leaves. The similar temperature response of *g*_m_ and *A*_max_ is expected as *g*_m_ positively affects *A*_max_. The similar temperature response of *g*_bs_ and *A*_max_ found here is unexpected, as *g*_bs_ negatively affects *A*_max_ in C_4_ leaves. Nevertheless, given the uncertainties in all input parameters we used, our result on the temperature response of *g*_bs_ (Fig. 6) should be considered as tentative, and, as already stated, needs confirmation by other independent techniques. Our sensitivity analysis showed that the estimated temperature response of *g*_bs_ depended only slightly on the assumed temperature response of *g*_m_ (Supplementary Fig. S3), but more on Rubisco and PEPc kinetic parameters (Fig. 7). Using extreme values of some kinetic parameters occasionally caused a change from an optimum response to an accelerating response to temperature (Fig. 7). The *g*_bs_ temperature response rarely depends on *g*_m_ parameters but more on Rubisco and PEPc kinetic parameters; this is expected from the model of von Caemmerer and Furbank (1999), because the calculation of *C*_c_ is minimally affected by *g*_m_ through *C*_m_, and *V*_c_ depends strongly on *g*_bs_ and *V*_cmax_. However, those kinetics parameters to which the estimated temperature response of *g*_bs_ is very sensitive were either measured for maize (e.g. *K*_mC25_, *K*_mO25_,and γ*_25_, reported by Cousins *et al.*, 2010) or were relatively conserved among C_4_ species (*E*_Vcmax_ and *E*_γ*_; see above). Also uncertainties related to a set of PEPc kinetics parameters as a whole had little impact on *g*_bs_ estimates (Supplementary Table S1), although individual parameters such as *D*_Vpmax_ and *S*_Vpmax_—when varied independently—strongly influenced the estimated *g*_bs_ (Fig. 7). Despite these uncertainties of the input parameters, the obtained *g*_bs_–*T*_leaf_ relationship between 13.5 °C and 39.0 °C followed either a peaked or non-peaked Arrhenius pattern (Figs 6, 7). Below 35 °C, the estimated *g*_bs_ almost exclusively increased monotonously with increasing temperature (Fig. 7). The activation energy estimate based on Equation 3 for our estimates of *g*_bs_ in Fig. 6 within 35 °C was ~74.45 kJ mol^−1^ for our study, and its corresponding *Q*_10_ value was ~2.74.

The *Q*_10_ factor for diffusion of CO_2_ in water is ~1.25 (Bernacchi *et al.*, 2002). Our higher *Q*_10_ value for *g*_bs_ suggests the possibility that some proteins/enzymes are involved in inter- and intracellular CO_2_ diffusion in C_4_ leaves. One candidate is CA, which facilitates the CO_2_ diffusion rate (Badger and Price, 1994). CA has often been considered to play a role in mediating *g*_m_ in C_3_ species (e.g. Bernacchi *et al.*, 2002). Its role for *g*_m_ in C_4_ leaves is justified since in C_4_ plants CA is mainly found in the cytosol alongside PEPc (Ludwig *et al*., 2011), and CA activity is temperature dependent (Boyd *et al.*, 2015). This actually gives indirect support to the use of the modified Arrhenius response for the temperature effect on *g*_m_ in our analysis. The bundle-sheath cells may contain some amount of CA isoforms (Ludwig *et al*., 2011), seemingly in support of its potential mediating role for *g*_bs_ as well. The other candidate may be an aquaporin that increases the CO_2_ permeability of the cell membrane (Terashima and Ono, 2002). Genetic manipulation of specific aquaporins has been used to vary *g*_m_ in C_3_ species (e.g. Hanba *et al.*, 2004). Brautigam *et al.* (2010) showed that a 20-fold up-regulation in the abundance of an mRNA coding for an aquaporin was registered in a C_4_ species. Other temperature-dependent activities may also shape our observed *g*_bs_–temperature relationship. For example, the current model simply assumes that the rate of decarboxylation equals *V*_p_. Recently it has been suggested that maize leaves have mixed decarboxylation pathways (Furbank, 2011). Theoretical modelling by Wang *et al.* (2014) showed that engaging the mixed pathways could decrease the need to maintain a high concentration gradient of metabolites between mesophyll and bundle-sheath cells, reconciling an earlier analysis of Sowinski *et al.* (2008) that simple diffusion-driven transport of metabolites is not adequate to explain the metabolite exchange during C_4_ photosynthesis. The mixed decarboxylation pathways and associated cell to cell exchange of metabolites suggest it likely that PEPc carboxylation and the overall decarboxylation rates are not the same and have different temperature responses. In that case, such a difference will have been lumped with our estimated parameters for temperature response of *g*_bs_.

The temperature response of *g*_bs_, together with the temperature responses of *J*_atp_, Rubisco, and PEPc kinetic parameters, co-determined the temperature responses of leakiness ϕ (Fig. 8), and *V*_o_:*V*_c_ and *V*_c_:*V*_p_ ratios (Fig. 9). In line with the trends shown by theoretical modelling (von Caemmerer and Furbank, 1999) and experimental calculation (Kromdijk *et al.*, 2010; Pengelly *et al.*, 2010; Yin *et al.*, 2011; Ubierna *et al.*, 2013; Bellasio and Griffiths, 2014), the calculated ϕ declined sharply with increasing irradiance (Fig. 8a). However, ϕ at low irradiances even exceeded 1.0 when the temperature was above 30 °C (Fig. 8a). The extreme ϕ values >1 at strictly limiting irradiances and high temperatures resulted from relatively high mitochondrial respiration in bundle-sheath cells combined with relatively high photorespiratory rates (as indicated by relatively high *V*_o_:*V*_c_ ratios at low irradiances; Fig. 9a). As a result, the temperature response of ϕ did not vary with CO_2_ levels but depended strongly on the irradiance levels (Fig. 8c). The estimated ϕ at high irradiances and the average ϕ of various CO_2_ levels showed a flat peaked response to temperature (Fig. 8c), in line with the trend shown by von Caemmerer *et al.* (2014) with the carbon isotope method.

For an effective CCM, the *V*_c_:*V*_p_ ratio is expected to be <1.0. However, the *V*_c_:*V*_p_ ratio went up to >1.0 at low CO_2_, especially at high temperatures (Fig. 9d). The model of von Caemmerer and Furbank (1999) (see Equations A1 and A4 in Supplementary appendix A) predicts that the *V*_c_:*V*_p_ ratio is ≥1.0 only if *L≤*0.5*V*_o_+(*R*_d_–*R*_m_). This condition was met at our four lowest CO_2_ levels (*C*_i_ of 10–30 μmol mol^−1^) where *V*_o_ was high. The average ratios under normal irradiance and CO_2_ conditions (calculated by excluding those four low CO_2_ levels) responded to temperature. Overall, the *V*_o_:*V*_c_ ratio increased with temperature (insets in Fig. 9a, b), because increasing temperature favoured the RuBP oxygenation relative to carboxylation. However, this general temperature response of the *V*_o_:*V*_c_ ratio seemed to be modified by the temperature response of *g*_bs_. The temperature response of *g*_bs_ and its associated temperature response of leakiness (Fig. 8c) required *V*_p_ to vary accordingly, resulting in a non-linear response of the *V*_c_:*V*_p_ ratio to temperature (insets in Fig. 9c, d).

Our parameter estimates can be compared with previous literature reports. Our estimate of activation energy for *R*_d_, 41.9 kJ mol^−1^ (Table 2), is within the range of reports for *R*_d_ in C_3_ species (24.2–65.2 kJ mol^−1^; see review by Yin *et al.*, 2014) as well as the range reported for C_4_ species (28.2–57.8 kJ mol^−1^; Dwyer *et al.* 2007). Our estimate for *g*_m25_,1.33mol m^−2^ s^−1^ (Table 2), is within the range of the earlier estimated or suggested values for maize (Pfeffer and Peisker, 1998; Kromdijk *et al.*, 2010; Yin *et al.*, 2011; Barbour *et al.*, 2016), and is higher than that for C_3_ leaves (for which the maximum *g*_m25_ is ~0.6mol m^−2^ s^−1^; e.g. Loreto *et al.*, 1992). Our estimated *V*_pmax25_:*V*_cmax25_ ratio, 2.43, agrees with our earlier estimate 2.5 (Yin *et al.*, 2011). Biochemical measurements on this ratio were 2.1–2.5 (Pengelly *et al.*, 2010), 3.1 (Kubien *et al.*, 2003), or higher (Sage *et al.*, 1987). The leaf N content in our experiment was on average 1.1g N m^−2^. Our estimated *g*_bs25_, 2.87 mmol m^−2^ s^−1^ (Table 2), is within the values reported for maize, 1.5 mmol m^−2^ s^−1^ (Ubierna *et al.*, 2013), 0.37–2.35 mmol m^−2^ s^−1^ (Kromdijk *et al.*, 2010), and 0.82–4.64 mmol m^−2^ s^−1^ (Bellasio and Griffiths, 2014), and also agrees with our previous estimate for this leaf nitrogen level (Yin *et al.*, 2011). However, our estimates for the slope of *V*_cmax25_ and *V*_pmax25_ versus leaf nitrogen, 57.7 μmol and 140.3 μmol (g N)^−1^ s^−1^, respectively, are lower than the previous estimates [96.0 μmol and 242.2 μmol (g N)^−1^ s^−1^, respectively] by Yin *et al.* (2011). The difference between the two studies in glasshouse environments (i.e. ~1 month later in the present study than in the previous study of Yin *et al.*, 2011), cultivars used (‘Atrium’ versus ‘2-02R10074’), and leaf ranks for measurements (the seventh versus the eighth to ninth) might have caused this disparity. It has been shown that acclimation to growing light intensities affected photosynthesis parameters in maize (Kromdijk *et al.*, 2010; Bellasio and Griffiths, 2013) and other C_4_ species (Ubierna *et al.*, 2011).

Previously only one study (Kiirats *et al.*, 2002) has reported the temperature response of *g*_bs_; that is, *g*_bs_ increases almost linearly with increasing leaf temperature, in contrast to our result for the Arrhenius response. Their temperature was only up to 35 °C. If normalized to 25 °C, the response within temperatures up to 35 °C was still different between their study and ours (Supplementary Fig. S4): the activation energy estimate based on Equation 3 was ~24.92 kJ mol^−1^ for their study, lower than 74.45 kJ mol^−1^ for our study (see above). This may highlight species differences in temperature response of *g*_bs_, although the impact of methodological differences between the two studies and/or the impact of uncertainty in input parameter values for our study cannot be ruled out. Temperature response of *g*_m_ for C_3_ photosynthesis has recently been reported to vary greatly with plant species (Walker *et al.*, 2013; von Caemmerer and Evans, 2015). There is a need to investigate further whether or not species diversity exists with regards to temperature response of *g*_bs_ for C_4_ photosynthesis. To that end, comprehensive investigations on enzyme kinetic constants may need to be carried out across contrasting C_4_ species.

In short, our study demonstrates that in contrast to existing modelling assumptions, *g*_bs_ does vary with leaf temperature. Although the presented temperature response curve still needs to be confirmed by other independent techniques, our results provide a step forward to more accurate modelling of C_4_ photosynthesis and productivity, especially for maize, under changing climatic environments.

## Supplementary data

Supplementary data are available at *JXB* online.

Appendix A. Basic equations in the C_4_ photosynthesis model of von Caemmerer and Furbank (1999) and analytical solutions of the model as given by Yin *et al.* (2011).

Appendix B. Quantifying temperature dependence of diffusivities and solubilities of CO_2_ and O_2_ in water.

Appendix C. Model and data for describing PEPc-limited rates of photosynthesis within the initial section of *A*–*C*_i_ curves.

Table S1. Estimated values of *E*_Kp_, *V*_cmax25_, and *g*_bs_ at six temperatures when using three sets of *V*_pmax_ parameters.

Figure S1. The initial linear section of *A*–*C*_i_ curves of 2% O_2_ at six measurement temperatures.

Figure S2. Comparison between modelled and measured *A*–*I*_inc_ and *A*–*C*_i_ curves at six leaf temperatures under the condition of 21% O_2_.

Figure S3. Temperature response of bundle-sheath conductance, estimated using four contrasting temperature responses of mesophyll conductance.

Figure S4. Comparison of temperature response of bundle-sheath conductance normalized to 1.0 at 25 °C between Kiirats *et al.* (2002) for *Amaranthus edulis* and our study for maize.

Supplementary Data
