# Supplementary material for: Temperature response of bundle-sheath conductance in maize leaves
Source: J Exp Bot. 2016 Mar 11;67(9):2699–714. doi: 10.1093/jxb/erw104 (PMC4861018; doi:10.1093/jxb/erw104)
Supplement: Supplementary Data [file supp_67_9_2699__index.html]

Temperature response of bundle-sheath conductance in maize leaves — Temperature response of bundle-sheath conductance in maize leaves — Supplementary Data 

# Temperature response of bundle-sheath conductance in maize leaves

## Supplementary Data

Data files

- Supplementary\_Appendices\_A\_B\_C\_Table\_S1\_Figures\_S1\_S4.pdf - Supplementary Data
