# Supplementary material for: Temperature response of bundle-sheath conductance in maize leaves
Source: J Exp Bot. 2016 Mar 11;67(9):2699–714. doi: 10.1093/jxb/erw104 (PMC4861018; doi:10.1093/jxb/erw104)
Supplement: Supplementary Data [file supp_erw104_Supplementary_Appendices_A_B_C_Table_S1_Figures_S1_S4.pdf]

Supplementary Data

Supplementary appendix A. Basic equations in the C_4 photosynthesis model of von Caemmerer & Furbank (1999) and analytical solutions of the model given by Yin et al. (2011)

The basic equations of the C_4 model (von Caemmerer & Furbank 1999) for net photosynthesis rate (A) are:

$$A = V_p - L - R_m \tag{A1}$$

$$L = g_{bs}(C_c - C_m) \tag{A2}$$

$$C_m = C_i - A / g_m \tag{A3}$$

A can be written in terms of the Rubisco carboxylation rate (V_c) and oxygenation rate (V_o):

$$A = V_c - 0.5V_o - R_d = \frac{(C_c - \gamma_* O_c)x_1}{C_c + x_2 O_c + x_3} - R_d \tag{A4}$$

where x_1, x_2, and x_3 are intermediate constants or variables: for the enzyme (Rubisco)-limited rate, $x_1 = V_{cmax}$, $x_2 = K_{mC}/K_{mO}$, $x_3 = K_{mC}$; for the e^- transport-limited rate, $x_1 = (1–x)J_{atp}/3$, $x_2 = 7\gamma_*/3$ and $x_3 = 0$.

The O_2 partial pressure in eqn (A4), O_c, is described as

$$O_c = \alpha A/(u_{oc} g_{bs}) + O_m \tag{A5}$$

where O_m is the mesophyll O_2 partial pressure (which we set here the same as O_i, the intercellular air-space O_2 partial pressure). The variable u_{oc} in eqn (A5) represents the coefficient that lumps diffusivities and solubilities of CO_2 and O_2 in water (von Caemmerer & Furbank 1999), and its possible dependence on temperature is quantified in Appendix B.

In the context of the modified model, eqn (1) in the main text, V_p is described by:

$$V_p = \begin{cases} \dfrac{C_m V_{pmax}}{C_m + K_p} & \text{if } A < \min(A_{TE}, A_{TT}) \\ xJ_{atp}/2 & \text{if } A = \min(A_{TE}, A_{TT}) \end{cases} \tag{A6}$$

The analytical solutions for individual terms of eqn (1) in the main text for C_4-photosynthesis are different, depending on whether V_p is limited by enzyme activity or by e^- transport. If the rate is limited by e^- transport, the solution is quadratic. If the rate is limited by PEPc activity, the solution is cubic.

The quadratic solution to A_{TE} or A_{TT}

The standard quadratic expression for A (referring to either A_{TE} or A_{TT}, for which V_p is limited by e^- transport) is (Yin et al. 2011):

$$aA^2 + bA + c = 0$$

where $$A = (-b + \sqrt{b^2 - 4ac})/(2a)$$

$$a = x_2 g_m \alpha / u_{oc} - g_m - g_{bs}$$

$$b = g_m (C_i g_{bs} + V_p - R_m) + (x_3 + x_2 O_i) g_m g_{bs} + (x_1 \gamma_* + x_2 R_d) g_m \alpha / u_{oc} + (g_m + g_{bs})(x_1 - R_d)$$

$$c = -g_m (C_i g_{bs} + V_p - R_m)(x_1 - R_d) + g_m g_{bs} [x_1 \gamma_* O_i + R_d (x_3 + x_2 O_i)]$$

where V_p is given by $xJ_{atp}/2$. In this solution, x_1, x_2 and x_3 are defined according to the text below eqn (A4), depending on whether the rate of the C_3 cycle is limited by the Rubisco activity or by e^- transport.

The cubic expression and its solution to A_{EE} or A_{ET}

The standard cubic expression for A (referring to either A_{EE} or A_{ET}, for which V_p is limited by the activity of the enzyme PEPc) is (Yin et al. 2011):

$$A^3 + pA^2 + qA + r = 0$$

The coefficients p, q, and r are complex combinations of model parameters or variables

$$p = m/(g_m + g_{bs} - x_2 g_m \alpha / u_{oc})$$

$$q = n/(g_m + g_{bs} - x_2 g_m \alpha / u_{oc})$$

$$r = o/(g_m + g_{bs} - x_2 g_m \alpha / u_{oc})$$

in which m, n, and o are expressed as

$$m = d - (x_3 + x_2 O_i) g_m g_{bs} + (R_d - x_1)(g_m + g_{bs}) - (x_1 \gamma_* g_m + x_2 R_d g_m - x_2 k/g_{bs}) \alpha / u_{oc}$$

$$n = f + (x_3 + x_2 O_i) k + d(R_d - x_1) - g_m g_{bs} [x_1 \gamma_* O_i + R_d (x_3 + x_2 O_i)] + (x_1 \gamma_* + x_2 R_d) k \alpha / (u_{oc} g_{bs})$$

$$o = R_d [f + (x_3 + x_2 O_i) k] - x_1 (f - k \gamma_* O_i)$$

where d, f and k are expressed as

$$d = g_m [R_m - V_{pmax} - C_i (g_m + 2g_{bs}) - K_p (g_m + g_{bs})]$$

$$f = g_m^2 [C_i V_{pmax} + (C_i + K_p)(g_{bs} C_i - R_m)]$$

$$k = g_m^2 g_{bs} (C_i + K_p)$$

In this solution, x_1, x_2 and x_3 are defined according to the text below eqn (A4), depending on whether the rate of the C_3 cycle is limited by Rubisco activity or by e^- transport.

Three roots for the above cubic equation are:

$$A_1 = -2\sqrt{Q} \cos(\psi / 3) - p/3$$

$$A_2 = -2\sqrt{Q}\cos[(\psi + 2\pi)/3] - p/3$$

$$A_3 = -2\sqrt{Q}\cos[(\psi + 4\pi)/3] - p/3$$

where $$Q = (p^2 - 3q)/9$$

$$\psi = \arccos(U/\sqrt{Q^3})$$

$$U = (2p^3 - 9pq + 27r)/54$$

We found the root A_1 suitable for calculating either A_{EE} or A_{ET} under any combinations of C_i, I_{inc} and O_i.

Supplementary appendix B. Quantifying temperature dependence of diffusivities and solubilities of CO_2 and O_2 in water

The coefficient u_{oc} in eqn (A5) in Appendix A actually lumps:

$$u_{oc} = D_{O2}S_{O2} / (D_{CO2}S_{CO2}) \quad \text{(B1)}$$

where D_{O2} and D_{CO2} are the diffusivities for O_2 and CO_2 in water, respectively, and S_{O2} and S_{CO2} are their respective solubilities in water. u_{oc} at 25°C is 0.047 (von Caemmerer & Furbank 1999).

von Caemmerer & Evans (2015) provided an equation, their eqn (3), describing the decrease in S_{CO2} with increasing temperature, based on data at the site (http://en.wikipedia.org/wiki/Henry's_law). Here we re-formulate it using the standard Arrhenius equation normalized at 25°C as:

$$S_{CO2} = S_{CO2,25} \cdot e^{-\frac{19.95}{R}\left(\frac{1}{298} - \frac{1}{273+T}\right)} \quad \text{(B2)}$$

where the universal gas constant R = 0.008314 kJ K^{-1} mol^{-1}. The data provided at the same website also allows quantifying the decrease of S_{O2} with increasing temperature:

$$S_{O2} = S_{O2,25} \cdot e^{-\frac{14.13}{R}\left(\frac{1}{298} - \frac{1}{273+T}\right)} \quad \text{(B3)}$$

Based on the report of Frank et al. (1996), von Caemmerer & Evans (2015) provided an equation describing the increase of D_{CO2} with increasing temperature. We rewrite the equation here normalized at 25°C as:

$$D_{CO2} = D_{CO2,25} \cdot e^{\frac{16.90}{R}\left(\frac{1}{298} - \frac{1}{273+T}\right)} \quad \text{(B4)}$$

Data of Han & Bartels (1996) show that D_{O2} also increases with increasing temperature, from which we obtained:

$$D_{O2} = D_{O2,25} \cdot e^{\frac{9.45}{R}\left(\frac{1}{298} - \frac{1}{273+T}\right)} \quad \text{(B5)}$$

Combining eqns (B2-B5) into eqn (B1) gives:

$$u_{oc} = 0.047 \cdot e^{-\frac{1.63}{R}\left(\frac{1}{298} - \frac{1}{273+T}\right)} \quad \text{(B6)}$$

With such small value of activation energy for u_{oc} (i.e., -1.63 kJ mol^{-1}), the model predicts that u_{oc} decreases from 0.048 at 13.5°C to 0.046 at 39°C of our experimental temperature range.

Supplementary appendix C. Model and data for describing PEPc limited rates of photosynthesis within the initial section of A-C_i curves

The photosynthetic rate in the initial part of an A-C_i curve can be approximated by (von Caemmerer & Furbank 1999):

$$A = C_m V_{pmax}/(C_m + K_p) - R_m - g_{bs} C_m \qquad \text{(C1)}$$

where C_m is the level of CO_2 in the mesophyll cells. Because g_{bs} is low (0.0009 to 0.0065 mol m^{-2} s^{-1} in our cases, Fig. 6) and the CO_2 levels for the initial part of an A-C_i curve are also low (Fig. S1), the last term of the above equation is negligible.

The difference in C_m from C_i is described by: $C_m = C_i - A/g_m$ (see Eqn A3). Combining this equation with Eqn (C1) yields a model:

$$A = \left(g_m(C_i + K_p) + V_{pmax} - R_m - \sqrt{[g_m(C_i + K_p) + V_{pmax} - R_m]^2 - 4g_m[C_i V_{pmax} - R_m(C_i + K_p)]}\right)/2 \qquad \text{(C2)}$$

The advantage of this model, eqn (C2), compared with the one described in the main text $dA/dC_i = K_p V_{pmax}/(C_i + K_p)^2$, is that it does not assume that g_m is infinite and the initial part of the A-C_i curves is strictly linear. Its disadvantage is that g_m has to be assumed beforehand. When we assumed a most likely value g_{m25} = 1.0 mol m^{-2} s^{-1} (Kromdijk et al. 2010), we estimated E_{Kp} when the model was combined with Eqn (3) for K_p and Eqn (4) for V_{pmax}. Using three sets of V_{pmax} parameters from Chinthapalli et al. (2003), Massad et al. (2007) and Boyd et al. (2015), the estimated E_{KP} based on data of the initial part of the A-C_i curves, and subsequently estimated V_{cmax25} and g_{bs} at six temperatures based on the full data set, are shown in Table S1.

Table S1 Estimated values (the standard error of the estimate in brackets) of E_{Kp}, V_{cmax25} and g_{bs} at six temperatures when using three sets of V_{pmax} parameters from Chinthapalli et al. (2003), Massad et al. (2007) and Boyd et al. (2015), respectively

	E_{Kp} (kJ mol^{-1})	V_{cmax} (μmol m^{-2} s^{-1})	g_{bs} (mmol m^{-2} s^{-1})					
			13.5°C	18°C	25°C	30°C	34°C	39°C
Chinthapalli et al. (2003)	66.3(4.1)	49.0(0.9)	0.98(0.39)	0.92(0.26)	2.56(0.36)	5.54(0.49)	6.40(0.48)	4.79(0.71)
Massad et al. (2007)	79.5(4.4)	49.3(0.9)	0.94(0.39)	0.98(0.27)	2.66(0.37)	5.93(0.51)	6.53(0.49)	4.39(0.69)
Boyd et al. (2015)	73.3(4.1)	49.0(0.9)	0.92(0.38)	0.94(0.26)	2.58(0.37)	5.61(0.49)	6.41(0.48)	4.52(0.69)

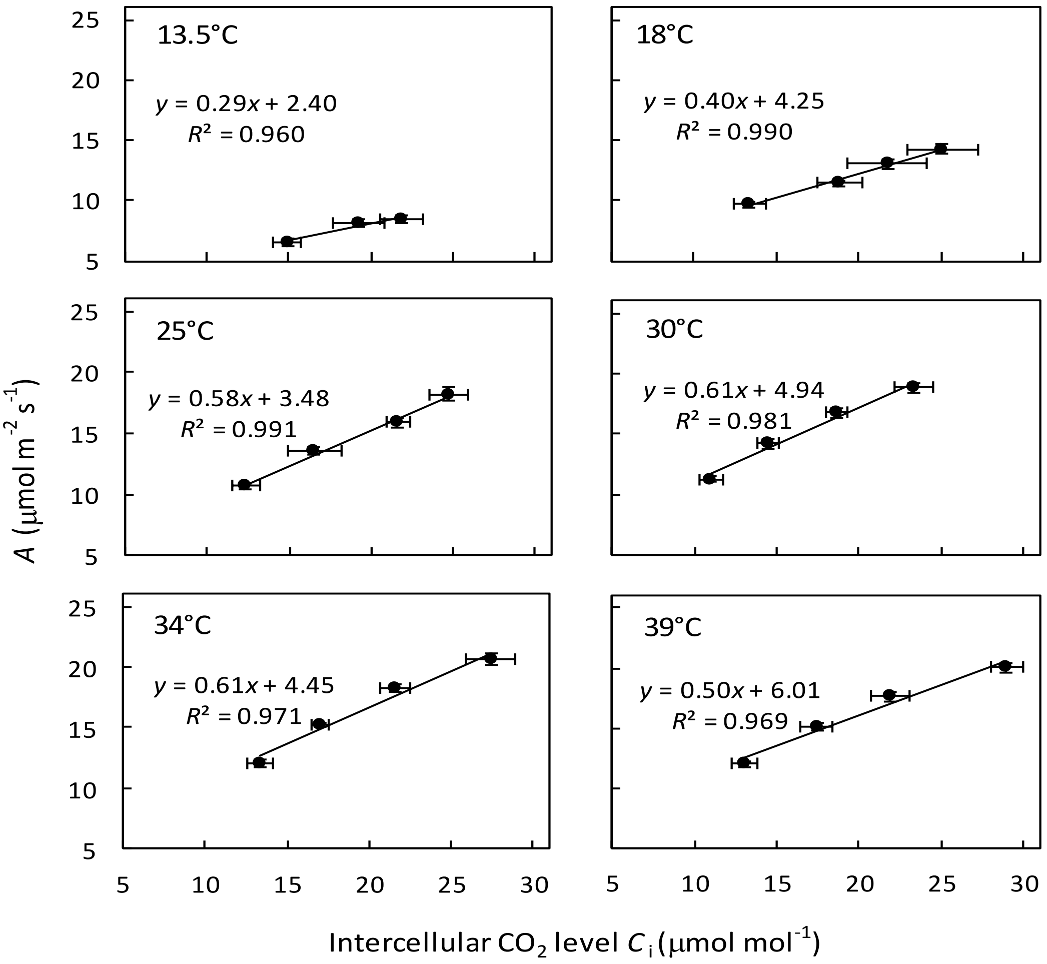

Figure S1 The initial linear section of A-C_i curves of 2% O_2 at six measurement temperatures. Bars represent standard errors of the mean of four replicated leaves (if larger than symbols).

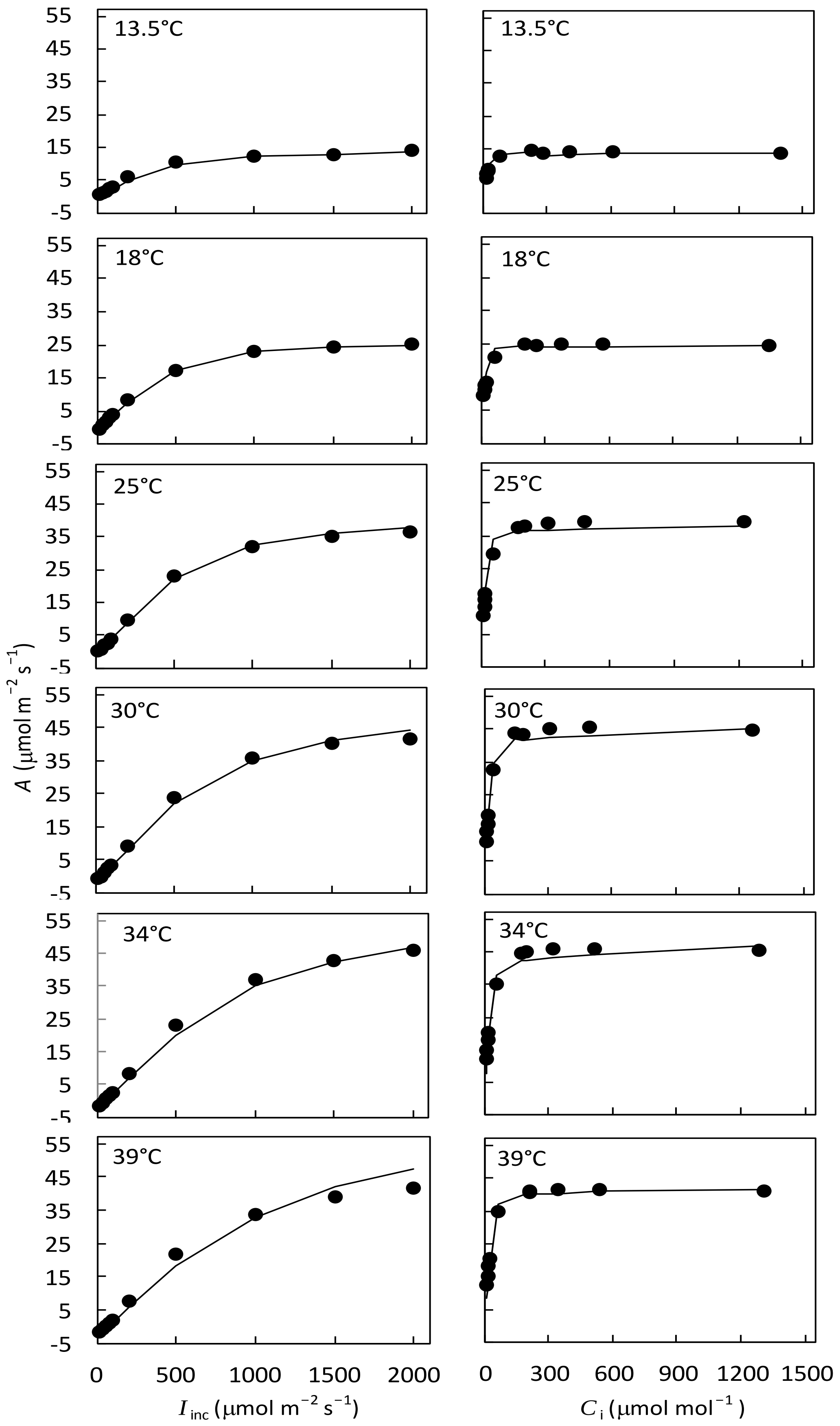

Figure S2 Comparison between modelled (curve) and measured (points) A-I_{inc} and A-C_i curves at six leaf temperatures under the condition of 21% O_2. The curves are drawn from connecting two nearby values calculated by the model.

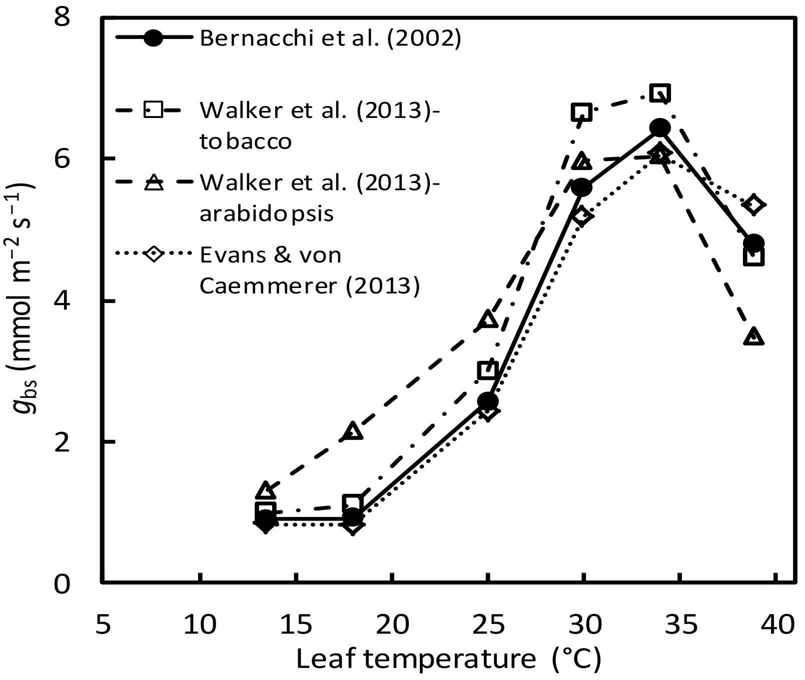

Figure S3 Temperature response of bundle-sheath conductance g_{bs}, estimated using four contrasting temperature responses of mesophyll conductance g_m as reported for C_3 photosynthesis by Bernacchi et al. (2002) for tobacco, Walker et al. (2013) for tobacco, Walker et al. (2013) for Arabidopsis, and Evans & von Caemmerer (2013) for tobacco.

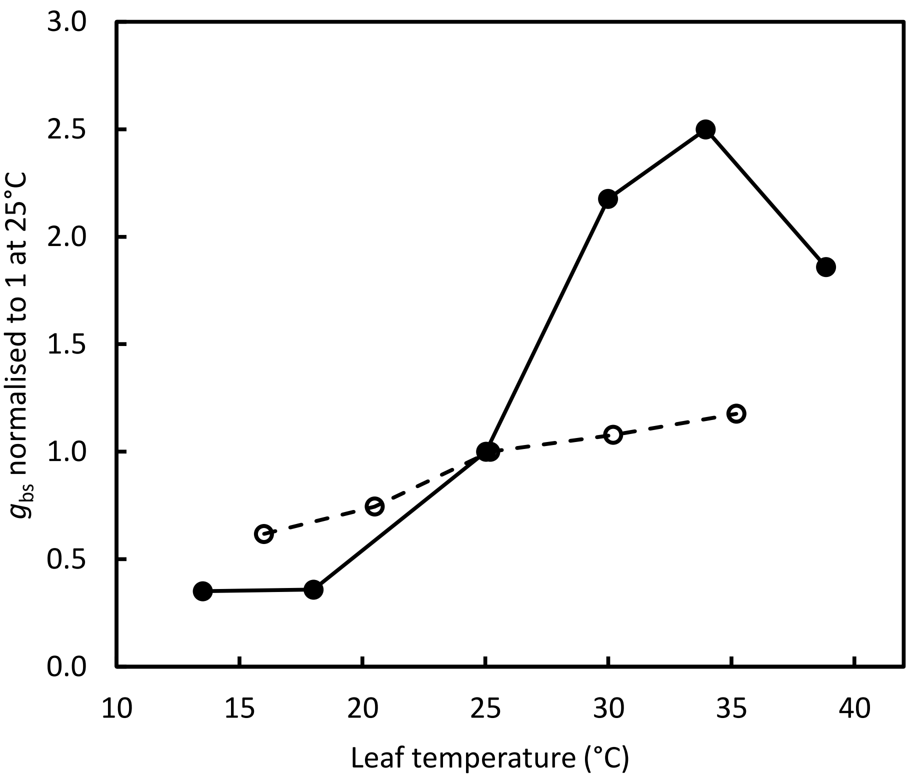

Figure S4 Comparison of temperature response of bundle-sheath conductance g_{bs} normalised to 1.0 at 25°C between Kiirats et al. (2002) for *Amaranthus edulis* (open symbols) and our study for maize (solid symbols based on data shown in Figure 6 in the main text).
